# White adipocytes in subcutaneous fat depots require KLF15 for maintenance in preclinical models

**DOI:** 10.1172/JCI172360

**Published:** 2024-07-01

**Authors:** Liang Li, Brian J. Feldman

**Affiliations:** 1 Department of Pediatrics, University of California, San Francisco (UCSF) School of Medicine, San Francisco, California, USA.; 2 Nutrition and Obesity Research Center, UCSF, San Francisco, California, USA.

**Keywords:** Cell biology, Endocrinology, Adipose tissue

## Abstract

Healthy adipose tissue is essential for normal physiology. There are 2 broad types of adipose tissue depots: brown adipose tissue (BAT), which contains adipocytes poised to burn energy through thermogenesis, and white adipose tissue (WAT), which contains adipocytes that store lipids. However, within those types of adipose, adipocytes possess depot and cell-specific properties that have important implications. For example, the subcutaneous and visceral WAT confers divergent risk for metabolic disease. Further, within a depot, different adipocytes can have distinct properties; subcutaneous WAT can contain adipocytes with either white or brown-like (beige) adipocyte properties. However, the pathways that regulate and maintain this cell and depot-specificity are incompletely understood. Here, we found that the transcription factor KLF15 is required for maintaining white adipocyte properties selectively within the subcutaneous WAT. We revealed that deletion of *Klf15* is sufficient to induce beige adipocyte properties and that KLF15’s direct regulation of *Adrb1* is a critical molecular mechanism for this process. We uncovered that this activity is cell autonomous but has systemic implications in mouse models and is conserved in primary human adipose cells. Our results elucidate a pathway for depot-specific maintenance of white adipocyte properties that could enable the development of therapies for obesity and associated diseases.

## Abstract 2

Targeting *Klf15* induces white adipocytes to burn more energy and have increased sensitivity to adrenergic stimulation.

## Graphical abstract

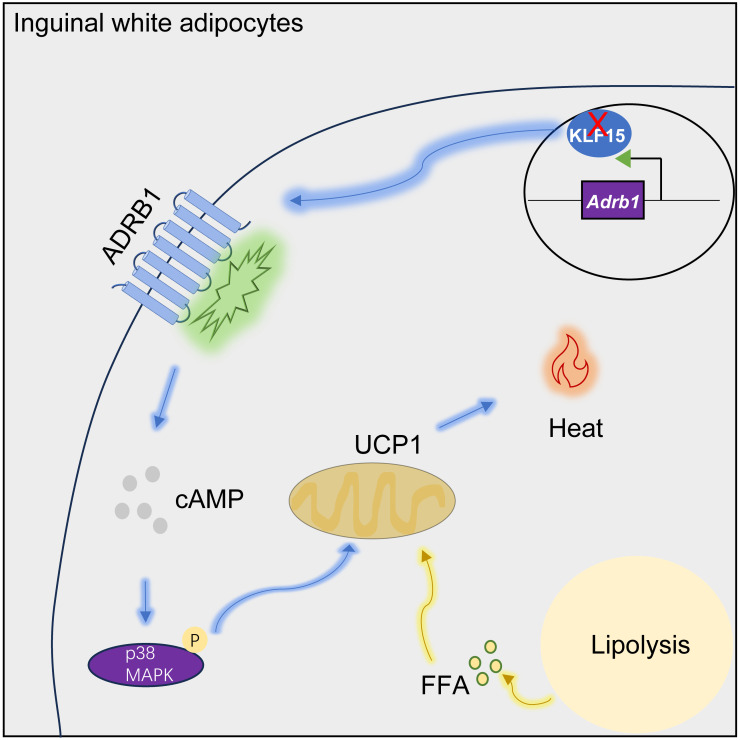

## Introduction

Adipocytes are the most prominent cell type in mature adipose tissue and, in addition to being involved in energy homeostasis, these cells receive and produce a variety of potent paracrine and endocrine signals in response to physiologic and extrinsic cues (1–4). Different adipose tissue depots have distinct development, maintenance, and influence on systemic metabolism. This is most obviously appreciated by comparing brown and white adipose tissue (BAT and WAT) depots (5–7). A substantial amount of WAT maturation occurs postnatally through the differentiation of tissue resident adipocyte progenitor cells (8). Conversely, mature BAT depots are present at birth in humans as well as in many other animals, including mice (9). Evolutionarily, the presence of BAT depots at birth is often attributed to the need for thermogenesis to maintain body temperature during the perinatal period, among other factors (10). Indeed, exposure to cold temperature is a potent physiologic activator of BAT — stimulating increased cellular oxygen consumption rates with uncoupled respirations — within brown adipocytes, which results in the production of heat (thermogenesis) (11, 12). This process is promoted, at least in part, by the β-adrenergic signaling induced by exposure to cold ambient temperatures (13).

The energy burning capability of BAT has garnered substantial interest not only because of the fascinating biology but also because the pathways regulating the generation and activation of BAT are potentially targetable for the development of therapeutics for obesity, a major health problem for millions of people (11, 14, 15). Yet, some enthusiasm for this approach has been tempered by the fact that humans appear to have few BAT depots at birth, and a further decline in the proportional amount of BAT compared with WAT depots occurs with aging (16).

However, more recently, it was discovered that some WAT depots, such as subcutaneous WAT, contain heterogenous types of adipocytes, including adipocytes that possess cellular and molecular features of brown adipocytes (17, 18). These adipocytes have been referred to as ‘brite’ (for ‘brown within white’) or ‘beige’ adipocytes (19–21). The precise origin of these cells remains incompletely understood, leaving it an open question whether they are derived from a distinct progenitor population, share a common progenitor with white adipocytes, or are converted from white adipocytes (19, 20, 22–26). Alternatively, and perhaps most likely, all these possible pathways occur and contribute to the composition of the WAT in vivo, with the origin of individual adipocytes being determined by variables in context, developmental stage, and stimulation that provokes the “beiging” of the WAT.

Given the substantial number of white adipocytes in most humans, it is enticing to contemplate targeting pathways that are critical to the maintenance of white adipocytes to promote therapeutic benefits. However, it remains poorly understood which factors are necessary to maintain white adipocyte properties. A major outstanding question remains: what are the context and depot-specific nature of these factors?

Kruppel-like factors (KLFs) are zinc finger motifs containing transcription factors that regulate development and systemic metabolism (27–29). There are 17 KLF family members, and dysregulation of KLFs results in diseases, including obesity and the development of diabetes (28). We (30, 31) and others (32, 33) have found that the Klf family member Klf15 affects adipose tissue, including regulating adipogenesis, lipid storage, and BAT. In this study, we describe our findings revealing a previously unrecognized and distinct role for KLF15 as a necessary factor for the maintenance of white adipocyte properties selectively in the subcutaneous WAT depot.

## Results

### Klf15 expression is higher in WAT compared with BAT and is suppressed by β-adrenergic signaling.

**Figure 1:**
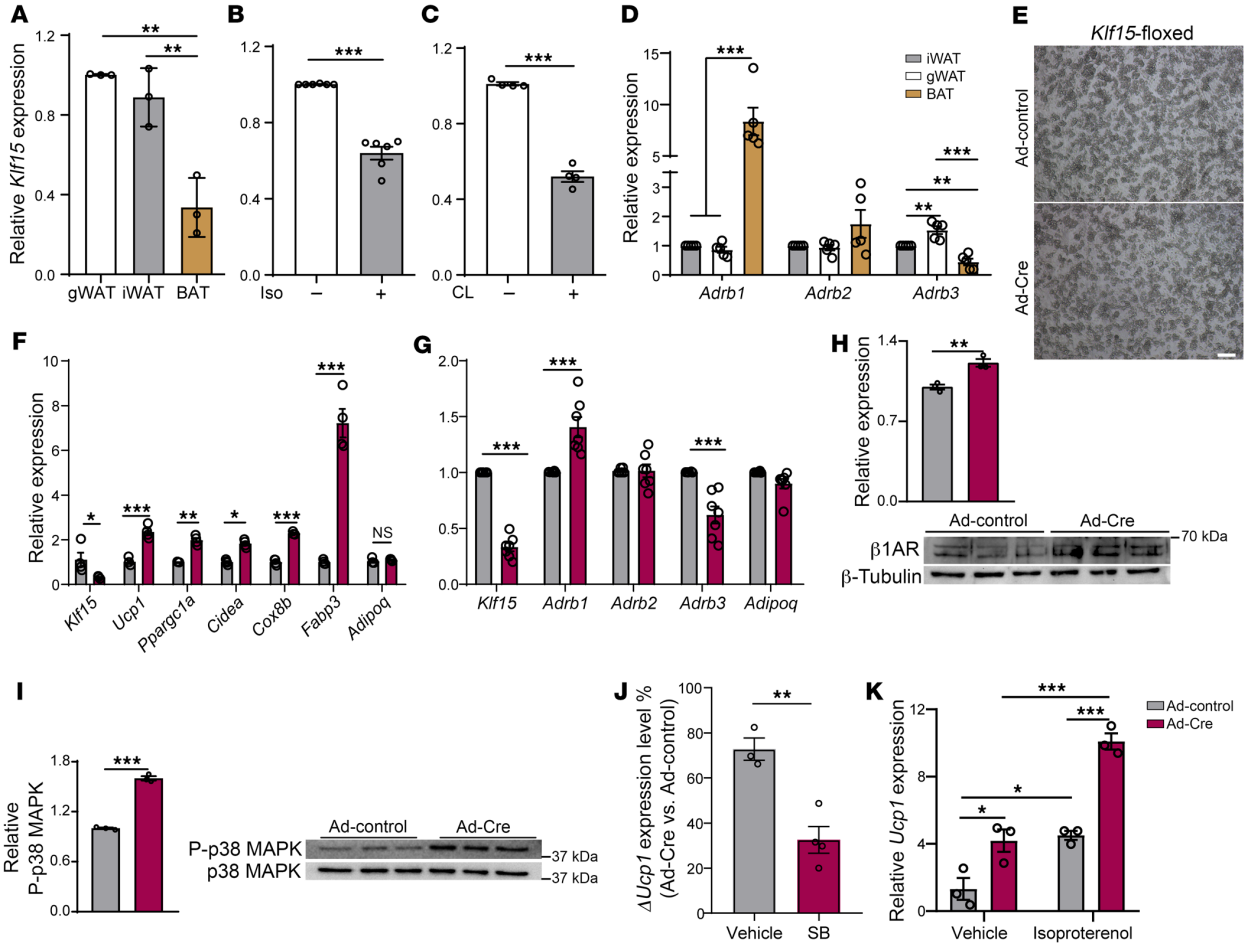
Acute modulation of *Klf15* expression in white adipocytes induces a beige fat expression profile. (**A**) RT-qPCR quantifying the expression levels of *Klf15* in adipocytes isolated from distinct fat pads. 1-way ANOVA, *n* = 3. (**B**) RT-qPCR quantifying the expression levels of *Klf15* in adipocytes after isoproterenol (Iso) treatment for 4 hours. Student’s *t* test, *n* = 6. (**C**) RT-qPCR quantifying the expression levels of *Klf15* in iWAT isolated from mice after they were injected with CL316243 (CL) (1 mg/kg/day) for 7 days. Student’s *t* test, *n* = 4. (**D**) RT-qPCR quantifying the relative expression levels of *Adrb1*, *Adrb2*, and *Adrb3* in iWAT, gWAT, and BAT with iWAT set to 1 for each litter. 1-way ANOVA, *n* = 5. (**E**) Light phase microscopy images of adipocytes from differentiated SVF harvested from the iWAT of *Klf15-fl/fl* mice and infected with adenovirus expressing Cre (Ad-Cre) or adenovirus control (Ad-control). Scale bar: 25 μm. (**F**) RT-qPCR quantifying the expression levels of thermogenic genes and panadipocyte marker *Adipoq*. Student’s *t* test, *n* = 4. (**G**) RT-qPCR quantifying the expression levels. Student’s *t* tests followed by Holm-Šidák correction, *n* = 7. (**H**) Immunoblots detecting and quantifying the relative levels of β1AR following acute *Klf15* deletion in adipocytes. Student’s *t* test, *n* = 3. (**I**) Immunoblots detecting and quantifying the relative levels of phosphorylation of p38 MAPK following acute *Klf15* deletion in adipocytes. Student’s *t* test, *n* = 3. (**J**) RT-qPCR quantifying the change in *Ucp1* expression levels with SB202190 pretreatment. Student’s *t* test, *n* = 3–4. (**K**) RT-qPCR quantifying the expression levels of *Ucp1* following acute *Klf15* deletion in adipocytes treated with Isoproterenol. 2-way ANOVA, *n* = 3. *P < 0.05, **P < 0.01, ***P < 0.001.

To elucidate the context-specific, as well as the most physiologically potent, activities of KLF15 in adipose tissue, we surveyed the expression levels of *Klf15* across 3 major types of adipose: visceral, subcutaneous, and intrascapular brown in WT mice. Strikingly, we found that the expression level of *Klf15* is approximately 75% lower in brown adipose tissue (BAT) than in white (WAT) (Figure 1A). These results suggest physiological implications for these differences, including raising the possibility of a requirement to downregulate *Klf15* levels for proper brown fat function. Brown fat responds to β-adrenergic signaling with the induction of thermogenesis (13), and this is a primary physiological function of this tissue. Therefore, we tested the impact of β-adrenergic stimulation on *Klf15* expression in adipose tissues. We found that exposing WAT to the β-adrenergic stimulant isoproterenol in tissue culture resulted in downregulation of *Klf15* expression (Figure 1B). To test if these results were relevant in vivo, we injected WT mice with the β-adrenergic agonist CL-316243 and discovered that this treatment results in an approximately 50% downregulation of *Klf15* expression levels in WAT (Figure 1C). There are 3 different adrenergic receptor family members (ADRB 1–3). Interestingly, the 3 adrenergic receptors have distinct properties as well as both ligand-dependent and constitutive activation (reviewed in ref. 34). We compared the expression levels of these receptors across the adipose types and found that *Adrb1* is the most differentially expressed in BAT compared with WAT (Figure 1D and Supplemental Figure 1A; supplemental material available online with this article; https://doi.org/10.1172/JCI172360DS1). An analogous pattern was observed when the expression levels of the adrenergic receptors were compared in human white and brown adipocytes (35). *Adrb1* is a particularly interesting, and perhaps distinct, adrenergic receptor; for example, overexpression of *Adrb1* in white adipocytes is sufficient to generate constitutive activity and mimics the effect of systemic infusion of agonists, even in the absence of additional agonists (36). These findings also indicate that increasing the expression level of the *Adrb1* alters white adipocytes in contexts where other adrenergic receptors are present.

### Deletion of Klf15 disrupts maintenance of white adipocyte properties and induces Adrb1 expression.

To begin to probe the physiological relevance of the above findings, we generated a *Klf15*-floxed mouse line using CRISPR/Cas9 to insert loxP sites surrounding exons 1–3 of the *Klf15* gene (*Klf15*-floxed). We then harvested white adipocytes from these mice and infected them with an adenovirus that expresses Cre recombinase (cleaving the loxP sites, knocking out *Klf15*) or control adenovirus (Figure 1E). Cre-infected adipocytes had lower expression levels of *Klf15* compared with controls (Figure 1F), demonstrating that Cre exposure properly results in efficient deletion of *Klf15* in the cells. We discovered that deletion of *Klf15* substantially induces the expression of genes that are critical to brown fat identity and function, including the expression of the canonical brown fat gene *Ucp1* (Figure 1F). These results are particularly striking because they support that there is plasticity in mature adipocytes, where core properties of white adipocytes can be modulated independently from adipogenesis.

When we measured the expression levels of β-adrenergic receptors in response to deletion of *Klf15*, we discovered that *Adrb1* was upregulated, while the other adrenergic receptors were unaffected or down regulated (Figure 1G). β1AR protein levels also increase with *Klf15* deletion (Figure 1H). These findings are intriguing, in part, because overexpression of *Adrb1* in subcutaneous white adipose tissue (iWAT) is sufficient to induce *Ucp1* expression and beiging of this white fat depot (36, 37). Further, β1AR is more sensitive to stimuli than other adrenergic receptors (38). Therefore, our results suggest a plausible pathway by which KLF15 could modulate the maintenance of white adipocytes and beiging in iWAT.

We next probed if the induction of brown fat genes that occurs after deletion of *Klf15* is mechanistically connected to the increased expression of *Adrb1*. Adrenergic receptor activation leads to increased phosphorylation of p38 MAP kinase which, in turn, induces *Ucp1* expression (39). To test if this pathway is stimulated by the deletion of *Klf15*, we monitored the levels of p38 phosphorylation. Indeed, deletion of *Klf15* leads to a marked increase in the levels of phosphorylated p38 in mature adipocytes (Figure 1I). Further, we found that inhibition of p38 MAP kinase suppresses the induction of *Ucp1* with *Klf15* deletion (Figure 1J).

As mentioned, β1AR activation can occur by both ligand-dependent and constitutive mechanisms. As no additional ligand was added to observe the induction of brown fat genes with *Klf15* deletion, our results raise the possibility that ligand-independent signaling was occurring because of the constitutive activation of the β1AR receptor. However, adrenergic receptor agonists are present in the cell culture medium (40), which could stimulate the pathway. Therefore, to integrate this question further, we assessed the effect of an adrenergic receptor antagonist. We discovered that the antagonist propranolol diminishes, but does not abolish, the induction of *Ucp1* expression in *Klf15*-deleted adipocytes (Supplemental Figure 1B). Reciprocally, we tested the effect of stimulation with an adrenergic receptor agonist. These experiments revealed that the addition of an agonist enhances the induction of *Ucp1* in *Klf15*-deleted adipocytes (Figure 1K). Together, these results support a model where the induction of *Adrb1* that occurs with *Klf15* deletion results in both ligand-dependent and constitutive β1AR activation in the mature adipocytes.

**Figure 2:**
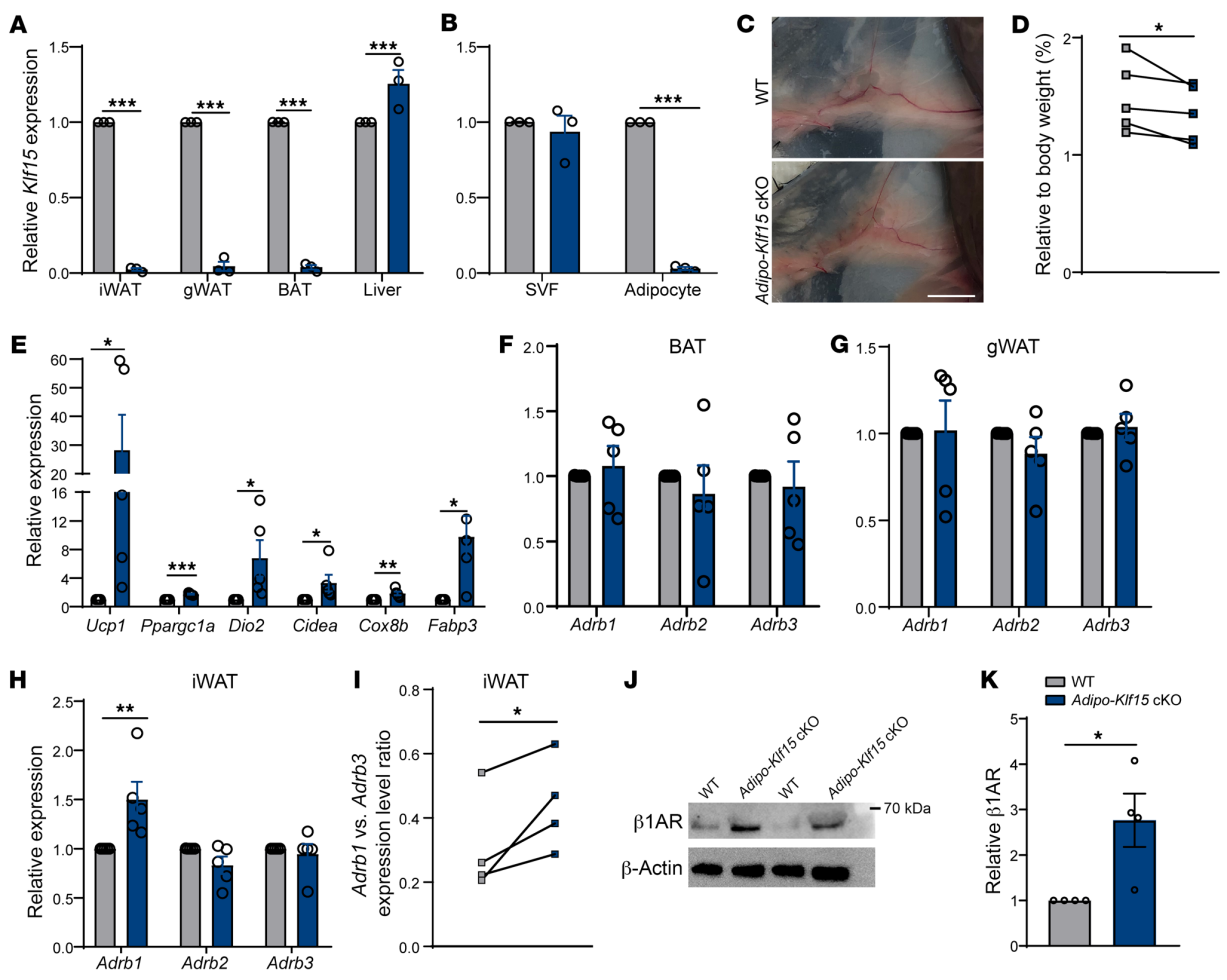
Adipocyte-specific *Klf15* KO promotes beige adipocyte formation in iWAT. (**A**) RT-qPCR quantifying the expression levels of *Klf15* in iWAT, gWAT, BAT, and liver from WT and *Adipo-Klf15* mice. Student’s *t* tests followed by Holm-Šidák correction, *n* = 3. (**B**) RT-qPCR quantifying the expression levels of *Klf15* in the SVF and adipocyte fraction of iWAT from WT and *Adipo-Klf15* mice. Student’s *t* test followed by Holm-Šidák correction, *n* = 3. (**C**) Representative images of in situ iWAT in WT and *Adipo-Klf15* mice. Scale bar: 5 mm. (**D**) Quantification of iWAT mass as a percent of body weight in WT and *Adipo-Klf15* littermates. Ratio paired *t* test, *n* = 5. (**E**) RT-qPCR quantifying the expression levels of thermogenic genes in iWAT of WT and *Adipo-Klf15* littermates. Student’s *t* test, *n* = 5. (**F**–**H**) RT-qPCR quantifying the expression levels of adrenergic receptors in iWAT, gWAT, and BAT from WT and *Adipo-Klf15* mice. Student’s *t* test followed by Holm-Šidák correction, *n* = 5. (**I**) RT-qPCR quantifying the expression levels of *Adrb1* versus *Adrb3* in iWAT in littermates of WT and *Adipo-Klf15* mice. 1-tailed ratio paired *t* test, *n* = 4. (**J**) Immunoblots detecting the levels of β1AR protein in iWAT from WT and *Adipo-Klf15* mice compared with the levels of β-actin controls. (**K**) Quantifying the relative protein level of β1AR. Student’s *t* test, *n* = 4. *P < 0.05, **P < 0.01, ***P < 0.001.

To investigate this activity of KLF15 selectively in mature adipocytes in vivo, we crossed the Klf15-floxed mice with transgenic mice expressing Cre recombinase under the control of the adiponectin promoter and regulatory elements (*Adipoq-Cre*). *Adipoq-Cre* mice have been verified to selectively express Cre in mature adipocytes (41), enabling conditional deletion of *Klf15* in mice that have both floxed *Klf15* and the *Adipoq-Cre* transgene (*Adipo-Klf15*–cKO mice) in their genomes. Indeed, we verified that *Klf15* is efficiently and selectively knocked-out in mature adipocytes and not in other tissues (Figure 2A) nor in the adipose stromal vascular fraction (SVF) (Figure 2B) of *Adipo-Klf15*–cKO mice. When we harvested the WAT from *Adipo-Klf15*–cKO mice, we noted that the iWAT had a browner appearance than iWAT from either *Adipoq-Cre* or *Klf15*-floxed mice (Figure 2C). In addition, the mass of the iWAT was less in *Adipo-Klf15*–cKO mice compared with littermate controls (Figure 2D). We discovered that the iWAT from *Adipo-Klf15*–cKO had substantially higher expression levels of multiple genes involved in brown fat identity and function (Figure 2E) and that upregulation of *Ucp1* was restricted to mature adipocytes isolated from the iWAT (Supplemental Figure 1C); we did not detect any induction of *Ucp1* in either visceral WAT (gWAT) or BAT (Supplemental Figure 1, D and E).

We also tested if there were analogous changes in the expression levels of the adrenergic receptors, as we discovered in our ex vivo experiments (Figure 1G). Consistent with our findings in tissue culture, the in vivo studies revealed that only *Adrb1* was selectively upregulated in the iWAT of *Adipo-Klf15*–cKO mice (Figure 2, F–H). As multiple factors, including external ones, can affect adrenergic signaling, to rigorously verify these results, we directly compared the ratio of expression levels of *Adrb1* to *Adrb3* in *Adipo-Klf15*–cKO mice against sex-matched littermates as individual paired replicates. The results of these orthogonal testing of the findings, which controls for any ‘crosstalk’ between Adrb1 and Adrb3 (42), confirmed that *Adrb1* expression in iWAT is selectively upregulated in *Adipo-Klf15*–cKO mice (Figure 2I and Supplemental Figure 1, F and G). Finally, we tested if the expression change translates into altered protein levels of β1AR. Using immunoblotting we established β1AR protein levels are increased in the iWAT of *Adipo-Klf1–*cKO mice (Figure 2, J and K).

Recently, noncanonical and adrenergic-independent thermogenic pathways have been described in adipocytes (43, 44). To evaluate if Klf15 affects these mechanisms, we measured the expression levels of genes involved in these pathways. We did not detect evidence that these pathways were altered by deletion of *Klf15* (Supplemental Figure 1H). These results further support that modulation of *Adrb1* by Klf15 is a primary mechanism for the beiging of mature adipocytes that we observed.

**Figure 3:**
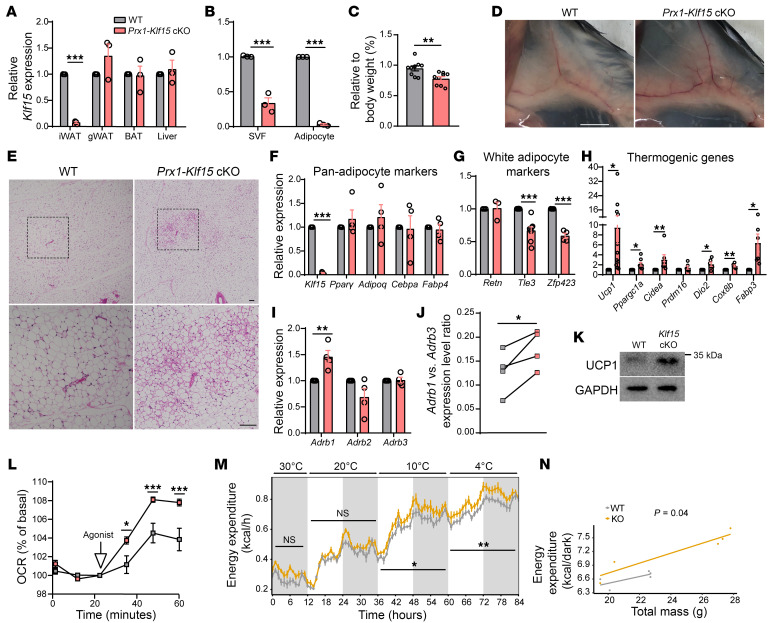
Enhanced beiging occurs in the iWAT of *Prx1-Klf15*–cKO mice. (**A**) RT-qPCR quantifying the expression levels of *Klf15* in iWAT, gWAT, BAT, and liver from WT and *Prx1-Klf15* mice. Student’s *t* tests followed by Holm-Šidák correction, *n* = 3. (**B**) RT-qPCR quantifying the expression levels of *Klf15* in the SVF and adipocyte fractions of iWAT from WT and *Prx1-Klf15* mice. Student’s *t* tests followed by Holm-Šidák correction, *n* = 3. (**C**) Quantification of iWAT mass as a percent of body weight in WT and *Prx1-Klf15* mice. Student’s *t* test, *n* = 9. (**D**) Representative images of in situ iWAT from female WT and *Prx1-Klf15* mice. Scale bar: 5 mm. (**E**) Representative images of H&E stained histological sections of iWAT from female WT and *Prx1-Klf15* mice. Bottom images are magnified from the indicated square in the top images. Scale bar: 100 μm. (**F**–**H**) RT-qPCR quantifying the expression levels of (**F**) panadipocyte markers, *n* = 4 (**G**) white adipocyte markers, *n* = 3–8, and (**H**) thermogenic genes in iWAT*, n* = 6–11. Student’s *t* test followed by Holm-Šidák correction (**I**) RT-qPCR quantifying the expression levels of adrenergic receptors in iWAT from WT and *Prx1-Klf15* mice. Student’s *t* test followed by Holm-Šidák correction, *n* = 4. (**J**) RT-qPCR quantifying the expression levels of *Adrb1* versus *Adrb3* in iWAT from littermates of WT and *Prx1-Klf15* mice. 1-tailed ratio paired *t* test, *n* = 4. (**K**) Immunoblot detecting UCP1 and GAPDH protein levels in the iWAT of WT and *Prx1-Klf15* mice. (**L**) Time course quantification of OCRs of iWAT isolated from WT and *Prx1-Klf15* littermates after exposure to Xamoterol using a Seahorse Bioanalyzer. 2-way ANOVA, *n* = 5. (**M** and **N**) Whole-body heat generation (kcal) of mice undergoing cold exposure. (**M**) 2-way ANOVA, (**N**) ANCOVA, *n* = 5. *P < 0.05, **P < 0.01, ***P < 0.001.

Together, our results indicate that deletion of *Klf15* in the *Adipo-Klf15*–cKO mice disrupts maintenance of white adipocyte properties and induces beiging selectively in the mature adipocytes within the iWAT depot. To investigate this further, as well as test the findings using an independent approach, we generated a second mouse line by crossing *Klf15*-floxed mice with transgenic mice expressing Cre under the regulation of *Prx1* promoter and regulatory element (*Prx1-Cre*). We (45) and others (46) previously demonstrated that this *Prx1-Cre* transgenic mouse line efficiently and preferentially targets the iWAT fat depot adipocyte progenitor cells over gWAT. Indeed, we found that mice generated by this cross that have both floxed *Klf15* and the *Prx1-Cre* transgene (*Prx1-Klf15* cKO) have substantially lower levels of *Klf15* expression selectively in the iWAT compared with other tissues and with littermate controls (Figure 3, A and B). In addition, the iWAT depots specifically weigh less, are browner, and have smaller adipocytes than iWAT depots in control mice (Figure 3, C–E and Supplemental Figure 2, A and B). While total body weights were not significantly different (Supplemental Figure 2, C and D), this may have been affected by the observed adipose depot–restricted effects combined with variable compensatory changes in food consumption (Supplemental Figure 2E).

Expression profiling revealed that the levels of panadipocyte-expressing genes were similar in *Prx1-Klf15*–cKO mice compared with littermate controls (Figure 3F). Notably, the *Prx1-Klf15*–cKO iWAT has decreased expression of some white adipocyte marker genes (Figure 3G) as well as significantly higher expression levels of multiple brown fat marker genes (Figure 3H). Further, the expression level of *Ardb1*, but not *Ardb2* or *Ardb3*, is upregulated in *Prx1-Klf15*–cKO iWAT as quantified in absolute levels (Figure 3I) as well as relative to *Adrb3* expression in littermate controls (Figure 3J). Immunoblotting confirmed that these expression differences translate into higher protein levels of UCP1 and β1AR in *Prx1-Klf15*–cKO iWAT (Figure 3K and Supplemental Figure 2F).

Having established, using 2 mouse model systems, that deletion of *Klf15* in iWAT induces β1AR and disrupts mature white adipocyte maintenance, we next investigated the functional implications of these findings. We measured the energetics of primary iWAT harvested from *Prx1-Klf15* cKO compared with littermate controls in response to the selective β1AR agonist Xamoterol as well as the adrenergic agonist Isoproterenol. We discovered that iWAT with *Klf15* deletion had an enhanced response to agonist stimulation, resulting in substantially higher induced OCRs (Figure 3L and Supplemental Figure 2G).

The above results indicate that deletion of *Klf15* stimulates upregulation of the β1AR and results in adipocytes with enhanced responsiveness to adrenergic stimulation. We next investigated the systemic implications of these findings in vivo. By monitoring singly housed mice in metabolic cages, we found that *Prx1-Klf15* cKO mice have higher energy expenditure per total body mass compared with littermate controls, and expenditure becomes significantly further induced by acute cold exposure, a physiologically relevant stimuli (Figure 3, M and N). Of note, the *Prx1-Klf15*–cKO mice are also more competent at maintaining their body temperature during cold exposure, and this occurs without any change locomotor activity (Supplemental Figure 3, A and B). These findings demonstrate that, even though this activity of KLF15 is cell-autonomous and restricted to the iWAT depot in mice, deletion of *Klf15* in iWAT has physiological implications.

Our ex vivo studies revealed that adipocytes with *Klf15* deletion have increased *Adrb1* expression and enhanced adrenergic sensitivity. We also found *Adrb1* expression levels are increased in the iWAT of mice with *Klf15* deletion. To assess the in vivo implications of these findings, we administered adrenergic agonists to the mice and monitored the effect on energy expenditure. We discovered that the *Prx1-Klf15*–cKO mice, housed in a thermoneutral environment, have enhanced energy expenditure immediately following injections with agonists, consistent with increased sensitivity to adrenergic stimulation during the short half-lives of these drugs (Supplemental Figure 3, C–F). As with cold exposure, the increased energy expenditure was not caused by increased activity (Supplemental Figure 3G).

### KLF15 modulates adipocyte sensitivity to β-adrenergic stimulation.

**Figure 4:**
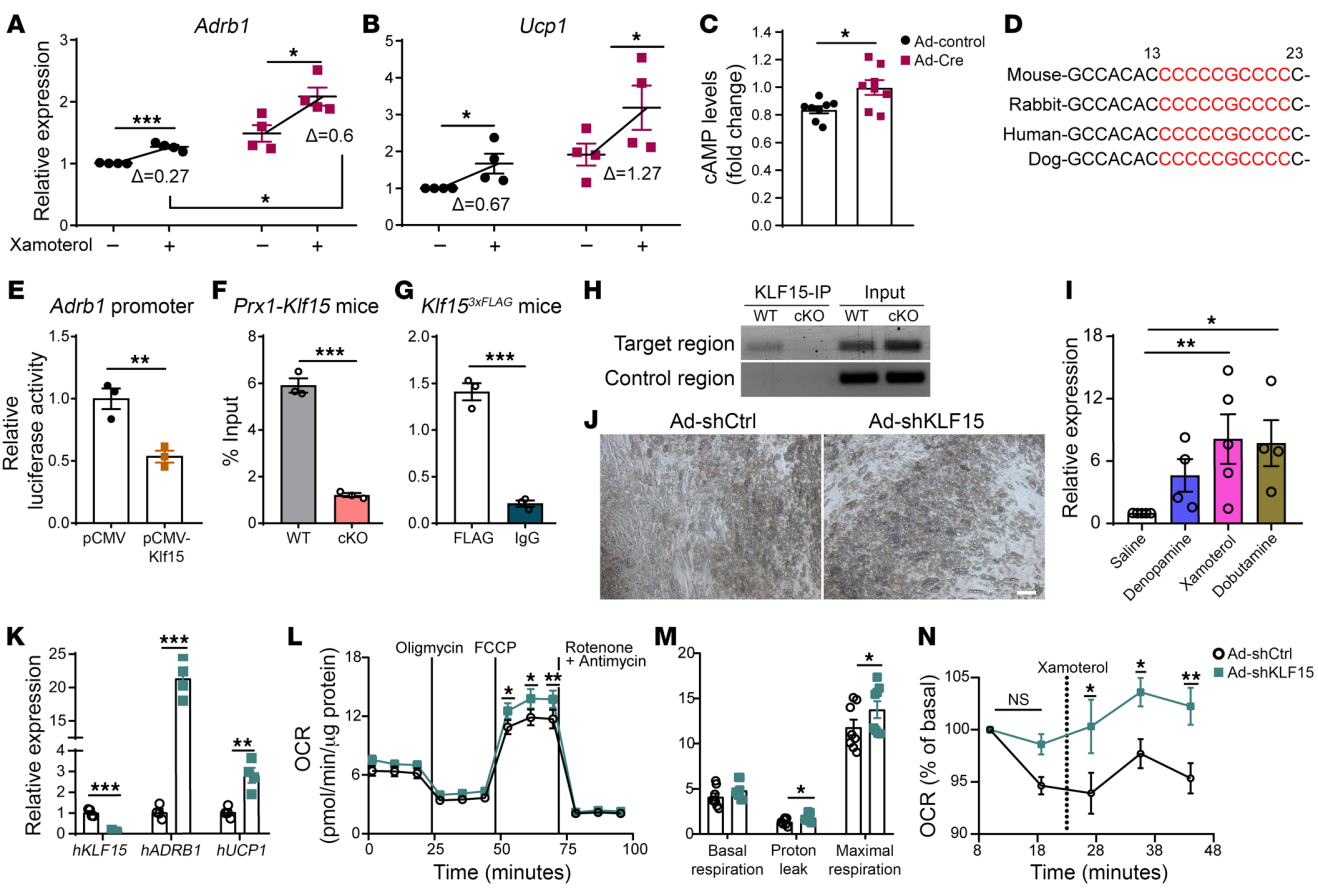
KLF15 regulates the expression of *Adrb1* in a pathway conserved in humans. (**A** and **B**) RT-qPCR quantifying the expression levels of *Adrb1* and *Ucp1* in adipocytes with or without Xamoterol treatment. Student’s *t* test, *n* = 5. (**C**) Quantifying of unstimulated intracellular cAMP levels in adipocytes with deletion of *Klf15*. Student’s *t* test, *n* = 5. (**D**) Conservation of a canonical KLF15 binding site sequence (red) identified in the mouse *Adrb1* gene. (**E**) Quantification of dual-luciferase assays on adipocytes transfected with the *Adrb1* promoter-driven firefly luciferase reporter construct and pCMV-Klf15 or control plasmid and normalized by Renilla bioluminescence, facilitated by cotransfected pRL-TK plasmid. Student’s *t* test, *n* = 3. (**F**) RT-qPCR quantifying the amount of immunoprecipitated DNA containing the putative KLF15 binding site located in *Adrb1* using a KLF15 antibody in iWAT adipocytes from WT and *Prx1-Klf15*–cKO mice. Student’s *t* test, *n* = 3. (**G**) RT-qPCR quantifying the amount of immunoprecipitated DNA containing the putative KLF15 binding site located in the *Adrb1* using the FLAG compared to IgG antibody in iWAT adipocytes isolated from *Klf15*^3xFLAG^ mice. Student’s *t* test, *n* = 3. (**H**) Image of PCR amplicons in an agarose gel of the putative KLF15 binding site (Target region) compared with amplification of the control region from the ChIP of adipocytes from WT and *Prx1-Klf15*–cKO mice with the KLF15 antibody. (**I**) RT-qPCR quantifying the expression levels of *Ucp1* in iWAT from mice injected with saline, Denopamine (10 μg/g/day), Xamoterol (8 ng/g/day), or Dobutamine (10 μg/g/day) for 7 days. 1-way ANOVA, *n* = 4–5. (**J**) Light phase microscopy images of human adipocytes. Scale bar: 25 μm. (**K**) RT-qPCR quantifying the expression levels of *hKLF15*, *hADRB1*, and *hUCP1* in human adipocytes infected by Ad-shCtrl or Ad-shKLF15. Student’s *t* test followed by Holm-Šidák correction, *n* = 4. (**L**) OCRs and (**M**) respiratory profile in Ad-shKLF15 and Ad-shCtrl infected human adipocytes quantified using a Seahorse Bioanalyzer, 2-way ANOVA *n* = 8. (**N**) Time-course of OCRs of human adipocytes after exposure to Xamoterol. 2-way ANOVA, *n* = 8–9. *P < 0.05, **P < 0.01, ***P < 0.001.

Having discovered that the deletion of *Klf15* in iWAT has systemic implications in vivo, we were motivated to further define the mechanisms driving this effect. Our results indicate that the β1AR receptor is upregulated in iWAT in both of our mouse models with *Klf15* deletions. Further, our findings demonstrate that this effect is cell-autonomous and occurs rapidly, as we detected marked changes in *Adrb1* following acute deletion of *Klf15* in cells (Figure 1G), suggesting that this change is proximal to, and potential directly caused by, KLF15 action. This model was particularly intriguing because adrenergic signaling stimulates a brown fat expression profile in iWAT and promotes cellular energy utilization in adipocytes through the induction of uncoupled respirations. Of note, prior work by others implicated β3AR as the primary adrenergic receptor expressed in white adipocytes in rodents (47). Our results raise the exciting possibility that targeting KLF15 could induce an alternative adrenergic input pathway for white adipocytes that promotes energy utilization. To test this model, we acutely deleted *Klf15* in white adipocytes using adenoviral infection of adipocytes. We then measured the expression levels of *Adrb1* and *Ucp1* both at the basal state as well as after stimulation with Xamoterol. We discovered that not only are basal levels of *Adrb1* and *Ucp1* expression higher in adipocytes after acute deletion of *Klf15* but also *Klf15*-deleted adipocytes have a more robust response to the β1AR agonist that is cell autonomous (Figure 4, A and B). Adrenergic signaling stimulates the induction of cAMP production within responsive cells (48). Therefore, we quantified cAMP levels in *Klf15*-deleted adipocytes. Indeed, we found that deletion of *Klf15* resulted in increased cAMP levels in the adipocytes (Figure 4C). These findings indicate that deletion of *Klf15* in white adipocytes enhances cellular sensitivity to adrenergic stimulation through the β1AR receptor.

### Adrb1 is a KLF15 target gene.

KLF15 functions as a transcription factor to both positively and negatively regulate the expression of genes by binding to a canonical DNA binding site sequence (Supplemental Figure 4A) in target genes (49). The rapid and substantial induction of *Adrb1* expression after *Klf15* knockdown suggested that expression of *Adrb1* could be directly modulated by KLF15. Interestingly, there is context-specificity to KLF15 action, as *Klf15* expression was found to stimulate expression of the α-adrenergic receptor *Adra1a* in models of nephric tubules (50). To probe deeper into the specific relationship between *Klf15* and *Adrb1* in white adipocytes, we acutely overexpressed *Klf15* in adipocytes using adenoviral infection; we discovered that overexpression of *Klf15* downregulates *Adrb1* expression (Supplemental Figure 4B), further suggesting that *Adrb1* is directly regulated by KLF15. Therefore, we scanned the promoter proximal region of the *Adrb1* gene and identified a KLF15 binding site sequence 23 bps downstream of the transcriptional start site of *Adrb1*. Importantly, we found that this putative binding site is highly conserved, including in humans (Figure 4D). We subcloned the endogenous *Adrb1* promoter and proximal region, containing this putative KLF15 binding site, into a promoterless luciferase reporter construct. We then transfected adipocytes with this luciferase reporter and cotransfected with either a *Klf15* expression vector or an empty control vector. Luciferase assays revealed that cotransfection with the *Klf15* expression vector inhibited *Adrb1* promoter activity compared with control infected cells (Figure 4E). Further, mutating the putative KLF15 biding site using site-directed mutagenesis of the *Adrb1* luciferase reporter construct (Supplemental Figure 4C) prevented KLF15 inhibition of *Adrb1* promoter activity (Supplemental Figure 4D), indicating that this binding site was necessary and sufficient for this KLF15 activity. Finally, we performed chromatin immunoprecipitation (ChIP) studies using primary iWAT isolated from WT mice as well as mice with a flag (3×) knocked into the *Klf15* gene (51) (*Klf15*^3xFLAG^) compared with iWAT from Klf15-cKO mice as a control. We performed ChIP on the putative KLF15 binding site in the *Adrb1* gene and found enriched occupancy at that site in both the iWAT of Klf15^3xFLAG^ mice and in WT mice (performed with anti-KLF15 and anti-FLAG, respectively) as well as compared with a distinct site located adjacent to the KLF15 binding site (Figure 4, F–H). Together, these data indicate that this site is a bona fide KLF15 binding site and response element in the *Adrb1* gene.

### KLF15 activity is conserved in primary human adipose cells.

We next tested for evidence that this pathway is conserved and relevant to humans. First, we injected WT mice with a panel of β1AR agonists, including ones that are FDA approved drugs for use in humans, and collected iWAT tissue. Strikingly, we discovered that these drugs provoked a substantial induction of *Ucp1* expression in the iWAT (Figure 4I). Next, we purchased human primary SVFs harvested from subcutaneous abdominal white adipose tissue biopsies. We differentiated these cells in culture and then knocked down human KLF15 (*hKLF15*) expression in the mature human adipocytes using adenoviral infection of Klf15 shRNA compared with control shRNA infection (Figure 4J). This approach resulted in a greater-than 80% knockdown of *hKLF15* in the human adipocytes (Figure 4K). Strikingly, acute knockdown of *hKLF15* resulted in a marked induction of both *hARDB1* and *hUCP1* in the primary human adipocytes (Figure 4K). Importantly, we found that induction of *hUCP1* was *hADRB1* dependent (Supplemental Figure 4E), implicating this pathway as the primary mechanism for the observed induction of *hUCP1*. In further support of this result, while knockdown of *hADRB1* blocked the induction of *hUCP1*, it did not alter the level of phosphorylated hormone-sensitive lipase (pHSL) (Supplemental Figure 4, F and G), indicating that the increased level of lipolysis with *hKLF15* knockdown observed by us (Supplemental Figure 4H) and others (32) is not the major driver of the induction *hUCP1* expression.

We next tested functional effects of *hKLF15* in human adipocytes by measuring cellular energetics. Using a Seahorse analyzer, we discovered that knockdown of *hKLF15* induces increased OCRs in human white adipocytes (Figure 4, L and M) without changing mitochondrial content (Supplemental Figure 4I); analogous to what we observed in our studies using the mouse models described above. Finally, we discovered that knockdown of *hKLF15* in human white adipocytes resulted in an enhanced induction of the OCR in response to adrenergic agonists Xamoterol and Dobutamine (Figure 4N and Supplemental Figure 4J and K). These results support that targeting *hKLF15* increases sensitivity to adrenergic stimulation in human adipocytes.

## Discussion

In this study, we discovered that Klf15 has a previously unrecognized depot-specific role in mature adipocytes in subcutaneous fat depots. We revealed that KLF15 is necessary to maintain white adipocyte characteristics in iWAT. Further, our results elucidate that the surprising mechanism of this action includes altering the adipocyte sensitivity to β-adrenergic stimulation. Our findings support that this pathway is conserved in humans.

We and others found that *Klf15* is expressed at relatively low levels in BAT and, reciprocally, *Adrb1* is robustly expressed in BAT at levels substantially above iWAT (Figure 1, A and D and Supplemental Figure 1A and (52)). We believe this defines the context-specific nature of the Klf15-Adrb1 pathway we identified in white adipocytes, where KLF15 regulation of *Adrb1* is a germane event that modulates white adipocyte maintenance. This, along with the use of distinct mouse models and assays that are not directly comparable, also likely explains differences observed in our study compared with studies focused on Klf15 in BAT (33). It is likely that increasing the already-high levels of *Adrb1* expression present in BAT has a relatively smaller impact than in the context of mature white adipocytes. It will be of future interest to identify if there are additional factors driving the high expression of *Adrb1* in BAT, but we believe the low levels of *Klf15* are likely relevant.

While understanding the mechanisms that regulate transcription of adrenergic receptors is of broad interest because of the potential implications for physiology as well as drug development, current knowledge in this area is limited. The findings of this study elucidate that *Adrb1* is a direct target gene of KLF15 in white adipocytes. Further, we reveal evidence that increased expression of *Adrb1* in white adipocytes results in enhanced β1AR signaling through a combination of tonic receptor activity and increased sensitivity to agonists. Together, our results define this previously unrecognized mechanism as critical to white adipocyte maintenance.

Within WAT depots, we also elucidated that the Klf15-Adrb1-Ucp1 pathway is context specific to iWAT. Subcutaneous and visceral adipose depots have numerous distinctions, including divergent implications for metabolic risk as well as the ability to beige (17, 53). Therefore, the context specificity we identified may be the result of fundamental distinctions in the cell populations. However, when we examined CHIP-Seq data sets from Roh et al., (54) we were intrigued to discover that the *Adrb1* locus undergoes epigenetic modifications in adipocytes in different contexts (Supplemental Figure 4L). Our studies identified *Adrb1* as a direct target gene of KLF15. It is tempting to speculate that these molecular mechanisms work together where different adipose depots and system signals are integrated by epigenetic differences as well as gating by KLF15 to generate context-specific responses in *Adrb1* expression.

Intriguingly, while ADRB3 appears to play a prominent role in rodent brown fat activation, *ADRB1* is the predominant adrenergic receptor in human BAT (55). Further, expression of *ADRB3* is not detectable in human WAT (35, 47). These observations likely, at least partially, explain why attempts to develop ADRB3 agonist as therapeutics for obesity in humans have not been successful (35). We identified KLF15 as a negative regulator of ADRB1 that is conserved in primary human adipose cells. These discoveries not only expand our understanding of adipose biology, including the plasticity of mature white adipocytes, but they also elucidate and define previously unrecognized pathways with plausible prospects for being more relevant, and therefore potentially more effective, therapeutic targets for humans than other approaches.

## Methods

### Sex as a biological variable.

Our study examined male and female animals, and similar findings are reported for both sexes.

### Animal models.

Mice were maintained on standard rodent chow diet with 12-hour light and dark cycles. All mice were in C57BL/6J background. Both male and female mice of various ages (young mice, 5–7 weeks old; adult mice, 8–16 weeks old) were used in these studies. *Klf15*-floxed mice were generated using CRISPR/Cas9 to insert loxP sites into the *Klf15* gene to surround exons 1–3. The *Prx1-Cre* mice (Stock No. 005584), and *Adipoq-Cre* (Stock No. 028020) mice were purchased from Jackson Laboratory. *Prx1-Klf15* cKO mice were generated from flox/flox, Cre/+ or flox/+, Cre/+ male mice cross with flox/flox, +/+ female mice. *Klf15*^3XFlag^ mice were generously provided by Saptarsi M. Haldar (University of California, San Francisco, California, USA).

For all in vivo studies, cohorts of at least 3 mice per genotype or treatment group were used, and experiments were repeated at least 3 independent times. Whole-body energy metabolism was measured using a Comprehensive Lab Animal Monitoring System (CLAMS) in the UCSF Nutrition and Obesity Research Center (NORC) core facility by a technician who was blinded to the genotypes. Age-matched mice at 7–8-weeks old were individually housed in rodent incubators at 30°C for 4 weeks before being placed in metabolic chambers for 5 days.

For the cold exposure studies, mice were housed in thermoneutral rodent incubators for 6 weeks prior to the experiments. These mice were then placed in metabolic chambers and exposed to a gradient of cold from 30°C to 10°C and then 4°C. Rectal temperatures were recorded 7 hours after mice were place in 10°C at 7 a.m.

For drug administration to mice, CL316243 (1 mg/kg/day, Sigma-Aldrich, C5976), Denopamine (8 ng/g/day, Sigma-Aldrich, D7815), Xamoterol (10 μg/g/day, Tocris, 0905) and Dobutamine (10 μg/g/day, Sigma-Aldrich, D0676), were injected intraperitoneally once a day.

### Cell culture.

Isolation of SVF from adipose depots were performed as previously described (30). Mouse adipocyte differentiation was induced by treating confluent preadipocytes with DMEM containing 10% FBS, 0.5 mM isobutyl methylxanthine (IBMX), 125 nM indomethacin, 2 μg/mL dexamethasone, 5 μg/mL insulin, 1 nM T3, and 1 μM rosiglitazone. Two days after induction, cells were switched to maintenance medium containing 10% FBS, 5 μg/mL insulin, 1 nM T3 and 1 μM rosiglitazone. Mouse cells were fully differentiated 6 days after inducing differentiation. To stimulate thermogenesis, differentiated cells were incubated with 10 μM isoproterenol for 4 hours or 1 μM Xamoterol for 3 hours before collecting samples. For inhibiting phosphorylation of p38 MAPK, adipocytes were pretreated with 15 μM SB202190 for 24 hours before collecting samples.

Human subcutaneous preadipocytes were purchased (PT-5020, Lonza) and differentiated according to the company’s instructions and as we previously described (56). Briefly, confluent preadipocyte were treated with Preadipocyte Growth Medium-2 (PGM-2TM) containing 10% FBS, 2 mM glutamine, IBMX (1:1000, PT-5020, Lonza), indomethacin (1:500, PT-5020, Lonza), dexamethasone (1:1000, PT-5020, Lonza), insulin (1:100, PT-5020, Lonza), 1 nM T3, and 1 μM rosiglitazone. Five days after induction, cells were switched to maintenance medium containing 10% FBS, 2 mM glutamine, insulin (1:100, PT-5020, Lonza), 1 nM T3, and 1 μM rosiglitazone for 5 days.

### Histology.

Freshly isolated adipose depots were fixed in 4% PFA, and then submitted for embedding, sectioning, and H&E staining at the UCSF Histology and Biomarker Core.

### Mitochondrial function and respiration.

For tissue respiration assays, 5 mg of adipose tissue were dissected from iWAT depots using a 2 mm surgical biopsy punch (96-1160, Sklar), placed into XF24 Islet Capture Microplates (101122-100, Agilent), and preincubated with Seahorse XF DMEM medium (103575-100, Agilent) with 25 mM glucose and 25 mM HEPES. The tissue was then rinsed again. After removal of the final rinse, running media (Seahorse XF DMEM medium with 25 mM glucose) was added and any remaining rinse media was removed. Finally, 480 μL of running media was added to all sample and control wells. After 3 cycles of basal measurements in the XF Extracellular Flux Analyzer (Seahorse Biosciences), 1 nM Xamoterol was injected into each well and measurements continued. O_2_ consumption was normalized to tissue weight and finally presented in the form of percentage of basic OCRs.

For human differentiated adipocyte, approximately 15,000 cells were seeded in each well of Seahorse XFe24 Cell Culture Microplates (100777-004, Agilent). Differentiated human adipocytes were infected with Adenovirus and then washed twice and preincubated in XF medium (containing 25 mM glucose, 2 mM glutamine, and 1 mM pyruvate) for 1 hour at 37°C without CO_2_. Oligomycin (1 mM), FCCP (4 mM), and Rotenone/Antimycin A (0.5 mM) were preloaded into cartridges and injected into XF wells in succession during the time course. For stimulation, 10 μM Xamoterol or 1 μM Dobutamine was injected after basal OCR measuring. OCR was measured and data were analyzed using Agilent Seahorse Analytics.

### RNA purification, reverse transcription, RT- PCR, and quantification of mitochondrial copy number.

Total RNAs from tissues were purified using QIAzol Lysis Reagent (QIAGEN) and pellet pestle (KIMBLE). Chloroform extraction of RNA was performed with tissue lysates depleted of lipid. RNAs in the aqueous phase were purified using RNeasy Mini Kit according to manufacturer’s instructions (QIAGEN). Total RNAs from cells were purified using RNeasy Mini Kit (QIAGEN). cDNA was synthesized using iScript cDNA Synthesis kit (Bio-Rad) according to the manufacturer’s protocol. RT–qPCR was performed using Biorad CFX96 system and TaqMan primers. Relative mitochondrial DNA content in human adipocytes was assessed by qPCR and calculated from copy number of the mtDNA-encoded MtCO1 gene and the nuclear DNA–encoded Ndufv1 gene.

### DNA constructs and transfection.

*Adrb1*-WT plasmid construct: The 5′ flanking sequence of the mouse *Adrb1* gene was amplified by PCR from mouse genomic DNA and subcloned into the pGL4.26 [Luc2/minP/Hygro] basic reporter gene vector (Promega) through DNA assembly (E2621G, NEB). The plasmid containing the regions between –216 to 40 from the transcriptional start site were obtained by annealing oligonucleotides 5′-TCAGAAACATGCTGAGGTCCC-3′ and 5′-GCCGAGCTGCGGAGG-3′. The *Adrb1*-Mut plasmid was generated from *Adrb1*-WT plasmid by performing site-directed mutagenesis (E0554S, NEB). Klf15 (NM_023184) mouse tagged (Myc-DDK) ORF was cloned into a pCMV6-Entry vector (MR206548, ORIGENE). All plasmids were purified using the HiPure Plasmid Midiprep Kit (K210004, Thermo Fisher Scientific). Differentiated adipocytes were transfected using Lipofectamine 3000 transfection reagent (Invitrogen) according to the manufacturer’s protocol.

### Luciferase reporter assay.

Transfections with 500 ng of plasmid DNA containing the *Adrb1* promoter-luciferase reporter constructs (*Adrb1*-WT or *Adrb1*-Mut) along with 100 ng of pRL-TK were performed in differentiated WT and *Prx1-Klf15* cKO adipocytes in 48-well plates. A combination of p3XFLAG-CMV 7.1 or pCMV-Klf15 with Adrb1-WT and pRL-TK were transfected into WT adipocyte cells. Forty-eight hours after transfection, luciferase activities were measured through GloMax Microplate Reader (Promega) by using the Dual-Luciferase Reporter Assay protocol.

### Immunoblotting.

For preparation of whole cell extracts from tissues, fresh or frozen tissue samples were homogenized using pellet pestle (KIMBLE) in 0.2 mL of protein extraction buffer (78510, Thermo Fisher Scientific) containing Complete Protease Inhibitor Cocktail EDTA-free (11836170001, Sigma-Aldrich). The homogenate was centrifuged at 4°C for 30 minutes at 15,000*g* and the supernatant portion containing the protein fraction was isolated using syringes and needles (38438, BD Biosciences), avoiding the lipid fraction. Protein lysates were loaded into 10% SDS-PAGE gels and transferred to a 0.2 μm nitrocellulose (1620112, Bio-Rad) or PVDF (1620177, Bio-Rad) membrane and immunoblotted using UCP1 antibody (1:1000, Ab10983; Abcam), ADRB1 antibody (1:1000, ab3442; Abcam), ADRB2 antibody (1:400, ab182136; Abcam), β-actin antibody (1:5000, A5441; Sigma- Aldrich), GAPDH antibody (1:300, sc-365062; Santa Cruz Biotechnology), p38 MAPK Antibody (1:1000, #9212, Cell Signaling Technology), Phospho-p38 MAPK (Thr180/Tyr182) Antibody (1:1000, #9211, Cell Signaling Technology), and β-Tubulin Antibody (1:1000, #2146, Cell Signaling Technology)

### Chromatin immunoprecipitation.

Chromatin immunoprecipitation was performed as previously described (57) with minor modifications. Briefly, adipose tissues were isolated from subcutaneous fat pads of 10–12 adult mice. White adipose tissues were minced with scissors in 5 mL DMEM containing 2% FBS. 600 μL 2% collagenase type 2 were added into minced adipose tissues, then placed at 37°C for 1 hour and 20 minutes. Samples were mixed by gently inverting tubes every 15 minutes. Nuclei extraction from the fixed adipocytes were performed using Covaris ultrasonicator (S220) at 4°C with a peak power of 75 W, a duty factor of 2%, and 200 cycles/burst for 2.5 minutes in a sonication tube (520135, Covaris) within 1 mL Farnham lab buffer. Isolated nuclei were resuspended in 1 mL shearing buffer supplemented with Complete Protease Inhibitor Cocktail EDTA-free (11836170001, Sigma-Aldrich) and transferred into a new sonication tube (520135, Covaris). Nuclei were sonicated at 4°C with a peak power of 50 W, a duty factor of 10%, and 200 cycles/burst for 8–10 minutes to shear the chromatin. The DNA was fragmented into approximately 200 bp segments (Agilent TapeStation 4200). Immunoprecipitation was performed with 10 μL of KLF15 antibody (ab2647, Abcam) on WT and *Prx1-Klf15*–cKO subcutaneous adipocytes, respectively; 6 μL FLAG antibody (F1804, Sigma-Aldrich) or 6 μL normal mouse IgG control (sc-2025, Santa Cruz Biotechnology) on *Klf15*^3xFLAG^ subcutaneous adipocytes overnight at 4°C. Antibody-bound chromatins were pulled down with Pierce ChIP-grade Protein A/G Magnetic Beads (26162, Thermos Fisher Scientific), washed, eluted, and reverse cross linked. DNA was extracted by phenol/chloroform/isoamyl alcohol and ethanol precipitated. The immunoprecipitated DNA from single experiment was quantified by qPCR using 3 different primers and regular PCR products were visualized after electrophoresis in 2% agarose gels (19E1004, 3:1 Super Sieve; IBSCI). The primer sequences are listed in supporting data file.

### Adenovirus and lentivirus infection.

SVF isolated from 5–7-week-old *Klf15*-floxed mice were equally seeded into 48-well plates. Differentiated cells were infected with Ad-Cre (Vector Development Laboratory [VDL]). Ad-GFP or Ad-Empty (VDL) were used as controls (termed Ad-Control). Differentiated human adipocytes were infected with sh-Ctrl or sh-Klf15 (Welgen). The hairpin sequence of sh-Klf15 was the same as previously reported (30). Cells were harvested 48 hours after infection for RT-qPCR. For the ADRB1 rescue experiment, adipocytes with *hKLF15* knockdown were infected with control shRNA (sc-108080, Santa Cruz Biotechnology) and β1AR shRNA (sc-29580-V, Santa Cruz Biotechnology) lentiviral particles.

### Lipolysis assay.

Adipocytes were washed and incubated in PGM-2TM medium containing 2 mM glutamine and 2% fatty acid–free BSA (A7030, Sigma-Aldrich) for 1 hour. Aliquots of medium were taken to assay for glycerol content using Glycerol-Glo (J3150, Promega) according to the manufacturer’s protocol. Glycerol content was normalized to total cellular protein content determined by a Bradford Assay (Thermo Fisher Scientific).

### Measurements of cAMP content.

Adipocytes infected with Ad-Control or Ad-Cre were used in cAMP-Glo Max Assays (V1681, Promega) according to the manufacturer’s protocol. Luminescence is inversely proportional to cAMP levels and was measured using a GloMax Microplate Reader (Promega) following the cAMP-Glo protocol.

### Statistics.

Statistical methods were not used to predetermine sample size. The experiments were not randomized, and investigators did not perform the experiments blinded. For comparisons between 2 independent groups, a student’s *t* test was used. For multiple comparisons ANOVA was performed with post hoc correction. 2-tailed *t* tests and 2-way ANOVAs were performed unless specifically designated otherwise in the figure legend. A *P* value < 0.05 was considered statistically significant. Statistical analysis and plotting for metabolic studies were performed using Calr. All other statistical analysis were performed with Prism Graph pad software.

### Study approval.

All mouse experiments in this study were performed under an approved IACUC protocol at the University of California, San Francisco (UCSF).

### Data availability.

Values for all data points in the graphs are reported in the Supporting Data Values file. Any additional information required to analyze the data reported in this paper is available from the lead contact upon reasonable request.

## Author contributions

BJF and LL were responsible for conceptualization, methodology, performing the investigation, and reviewing the manuscript. BJF acquired funding, supervised the project, and wrote the original draft of the manuscript.

## Supplementary Material

Supplemental data

Unedited blot and gel images

Supporting data values

## Version 1 07/01/2024

Electronic publication
