# Supplementary material for: White adipocytes in subcutaneous fat depots require KLF15 for maintenance in preclinical models
Source: J Clin Invest. 2024 Jul 1;134(13):e172360. doi: 10.1172/JCI172360 (PMC11213504; doi:10.1172/JCI172360)
Supplement: Supplemental data [file jci-134-172360-s189.pdf]

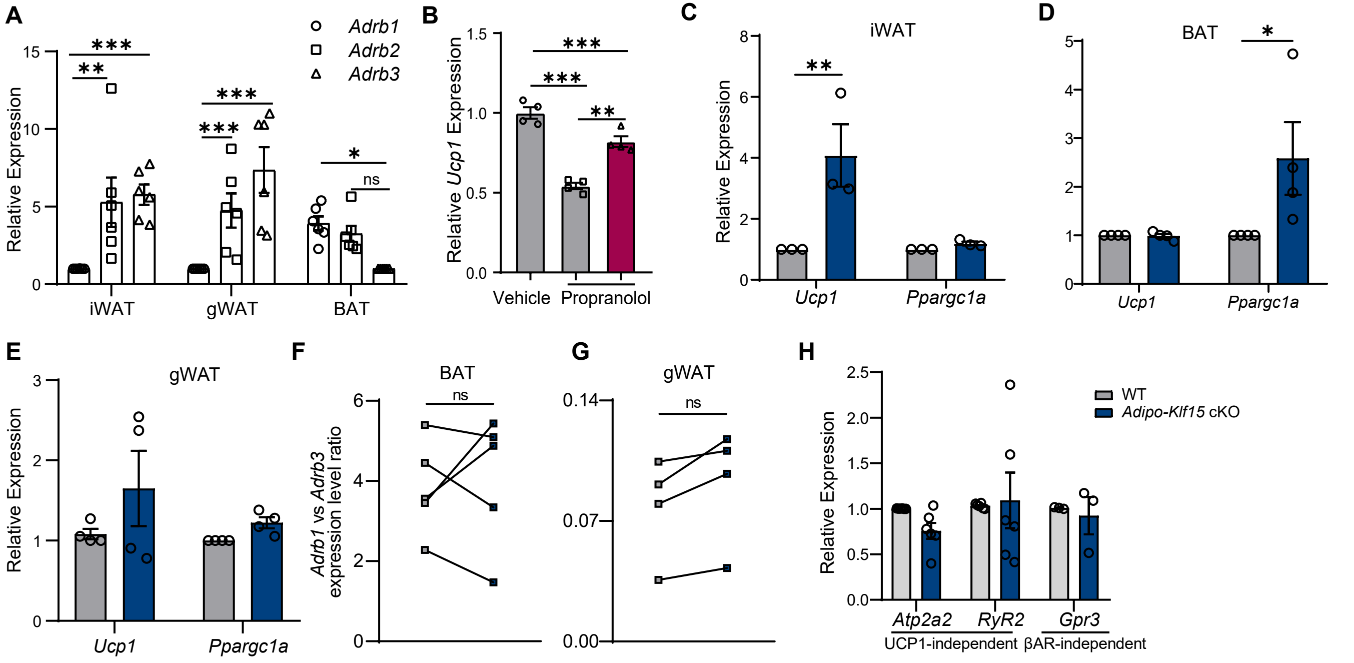

Supplemental Figure 1

Adipocyte-specific deletion of *Klf15* induces a thermogenic gene expression in the adipocyte fraction of iWAT. (**A**) RT-qPCR quantifying the relative expression level of *Adrb*1-3 in different adipose tissue depots. t tests followed Fisher's LSD test, n = 6. (**B**) RT-qPCR quantifying the expression levels of *Ucp1* after acute *Klf15* deletion in adipocytes treated with vehicle or propranolol for 24h. One-way ANOVA, n = 4. (**C**) RT-qPCR quantifying the expression levels of *Ucp1* and *Ppargc1a* in the adipocyte fraction of iWAT isolated from WT and *Adipo-Klf15* mice. Student's t test followed by Holm-Sidak correction, n = 3. (**D**, **E**) RT-qPCR quantifying the expression levels of thermogenic genes. Student's t test, n = 4. (**F**, **G**) Ratios of *Adrb1* vs *Adrb3* expression levels in BAT (**F**) and gWAT (**G**) from WT and *Adipo-Klf15* littermates, quantified by RT-qPCR. Ratio paired t test, n = 4-5.(**H**) RT-qPCR quantifying the expression levels of factors relevant to UCP1 and βAR independent pathways. Student's t test, n = 3-6. *p < 0.05, **p < 0.01,***p < 0.001.

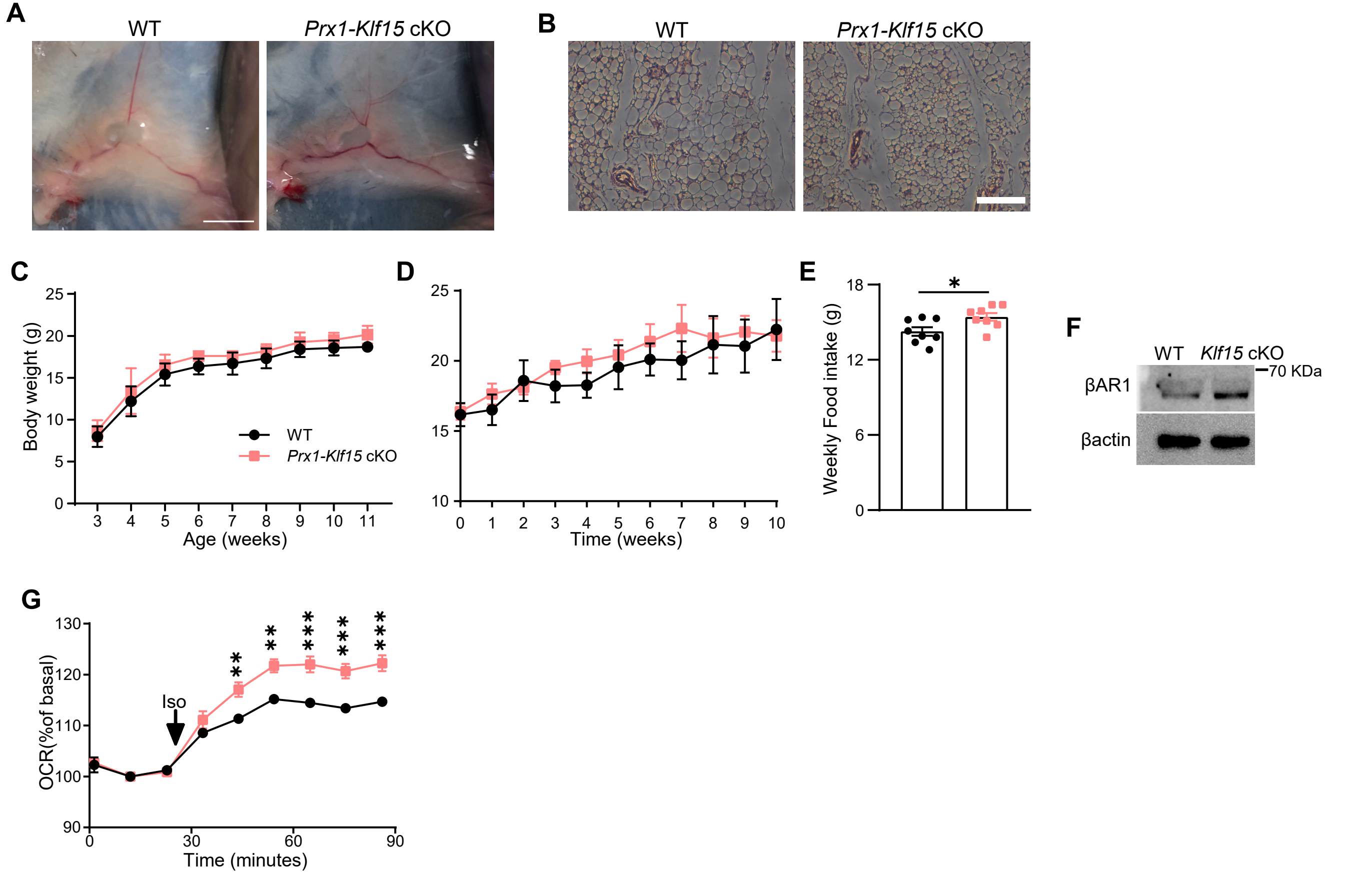

Supplemental Figure 2

Analysis of *Prx1-Klf15* mice. (**A**) Representative images of in situ iWAT from 6-weeks old male WT and *Prx1-Klf15* mice. Scale bar: 5 mm. (**B**) Representative images of H&E stained histological sections of iWAT from male WT and *Prx1-Klf15* mice. Scale bar: 1 mm. (**(C, D)** Body weights of female fed standard diets and HDFs. Two-way ANOVA n= 4-5. (**E**) Average weekly HFD intake of mice; each dot represent one-week food intake. Student's t test, n = 8. (**F**) Immunoblots detecting β1AR and βactin protein levels in the iWAT. (**G**) Time course quantification of OCRs of iWAT isolated from WT and *Prx1-Klf15* littermates after exposure to isoproterenol stimulation. Two-way ANOVA, n = 3-4. *p < 0.05, **p < 0.01, ***p < 0.001.

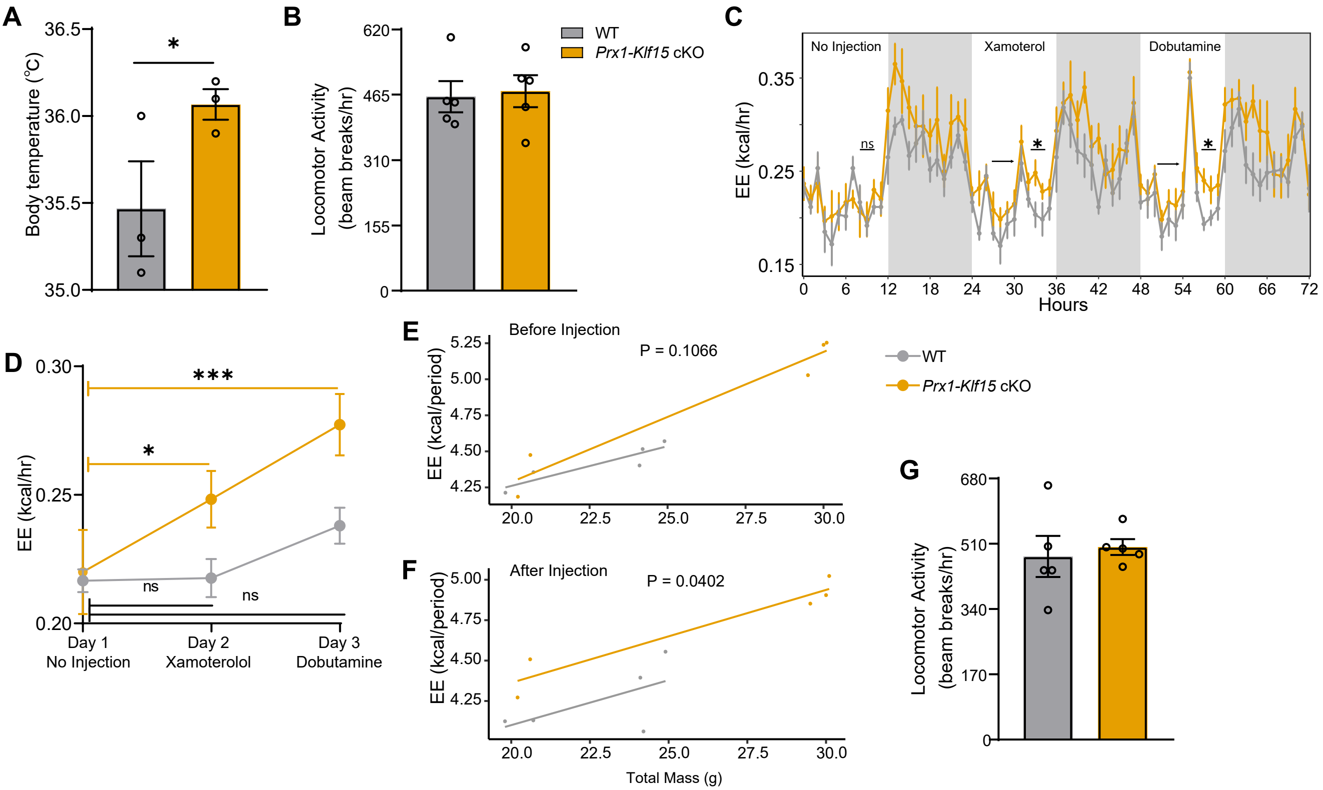

Supplemental Figure 3

Deletion of *Klf15* in iWAT increases systemic energy expenditure. (**A**) Rectal core body temperature of WT and *Prx1-Klf15* mice male mice maintained at 10 °C. One-tailed, t test, n = 3. (**B**) Quantification of the locomotor activity of mice during cold exposure. (**C**) Real-time monitoring of energy expenditure in WT and *Prx1-Klf15* mice in thermoneutral (30°C) cages and after agonist administration. (**D**) Quantification of the average change in energy expenditure during 5h after injection of agonist. Two-way ANOVA, n = 5. (**E, F**) Energy expenditure versus body weight in mice before (**E**) and after (**F**) agonist injection. ANCOVA, n = 5. (**G**) Quantification of the locomotor activity of mice in thermoneutral cages after agonist injection (time window: 30-64 in (**C**)). *$p < 0.05$. ***$p < 0.001$.

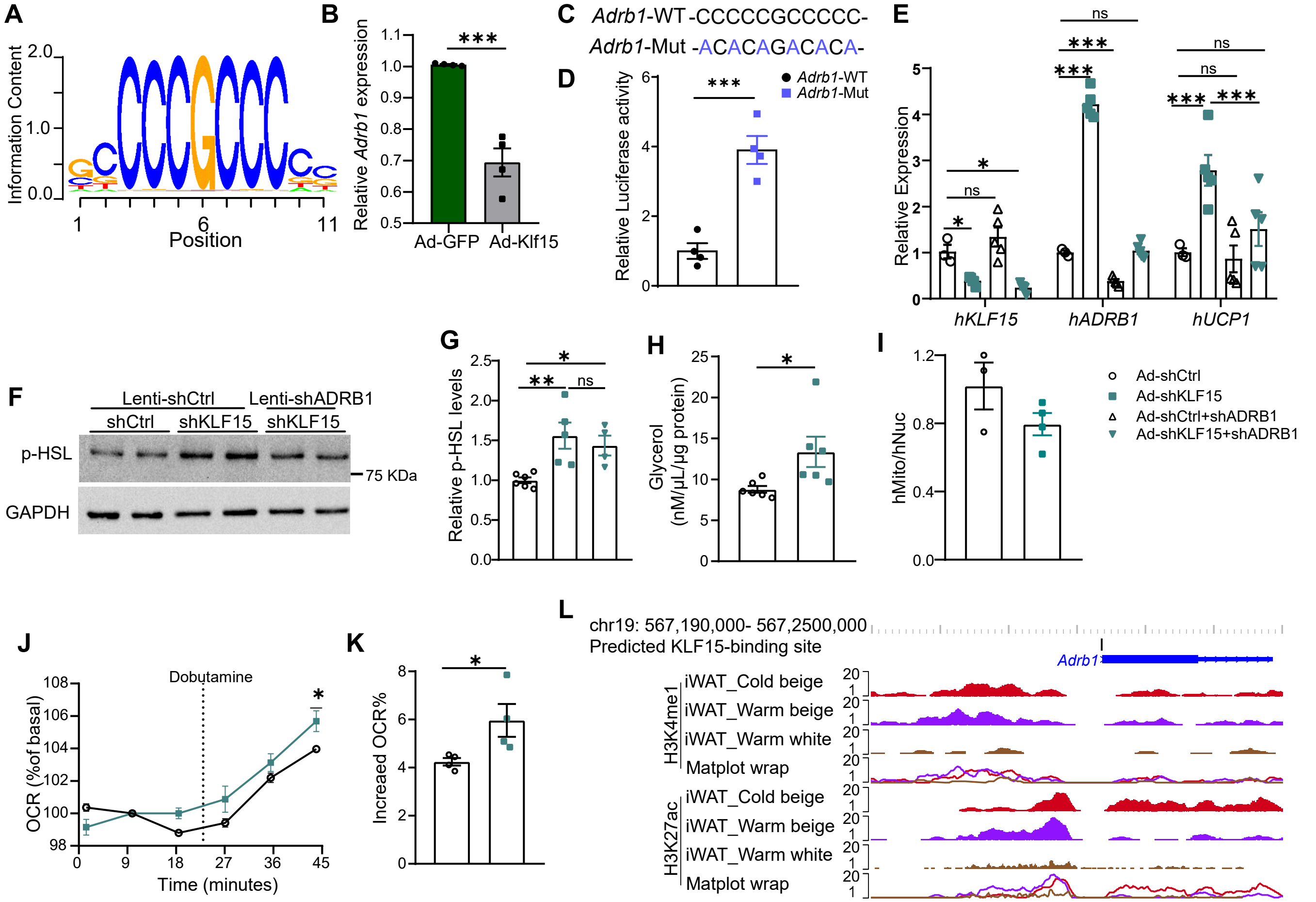

Supplemental Figure 4

KLF15 regulates *Adrb1* expression. (**A**) Position weight matrix of canonical KLF15 DNA binding motif. (**B**) RT-qPCR quantifying the expression levels of *Adrb1* from Ad-GFP and Ad-KLF15 infected adipocytes. Student's t test, n = 4. (**C**) DNA sequence of the wild-type and mutated *Klf15* DNA binding motif within the *Adrb1* luciferase reporter constructs. Nucleotides mutated using site-directed mutagenesis are depicted in purple. (**D**) Quantification of luciferase assays performed on adipocytes transfected with the *Adrb1*-WT or *Adrb1*-Mut promoter-driven luciferase reporter construct normalized using dual-luciferase with Renilla from the pRL-TK co-transfected plasmid. Student's t test, n = 4. (**E**) RT-qPCR quantifying expression levels in Ad-shRNA and Lenti-shRNA transduced human adipocytes. Student's t tests followed by Holm-Sidak correction, n= 3-5. (**F**) Immunoblots detecting the levels of pHSL protein from Ad-shRNA transduced human adipocytes. (**G**) Quantification of relative protein level of pHSL in human-derived adipocytes. One-way ANOVA, n= 4-6. (**H**) Quantification of glycerol released into the incubation media from human adipocytes. Student's t test, n = 6. (**I**) Relative mitochondrial (hMito) DNA content in transduced human adipocytes by RT-qPCR. Student's t test, n = 3-4. (**J**) Time course quantificating OCRs of human-derived adipocytes after exposure to Dobutamine. Two-way ANOVA, n = 4. (**K**) Quantification of increased OCR after dobutamine (1M) stimulation. Student's t test, n = 4. (**L**) H3K4me1 and H3K27ac ChIP-seq peaks at the *Adrb1* loci in different adipocyte contexts. *p < 0.05, **p < 0.01, ***p < 0.001.
