# Supplementary material for: White adipocytes in subcutaneous fat depots require KLF15 for maintenance in preclinical models
Source: J Clin Invest. 2024 Jul 1;134(13):e172360. doi: 10.1172/JCI172360 (PMC11213504; doi:10.1172/JCI172360)

Fig.1H

P-p38 MAPK

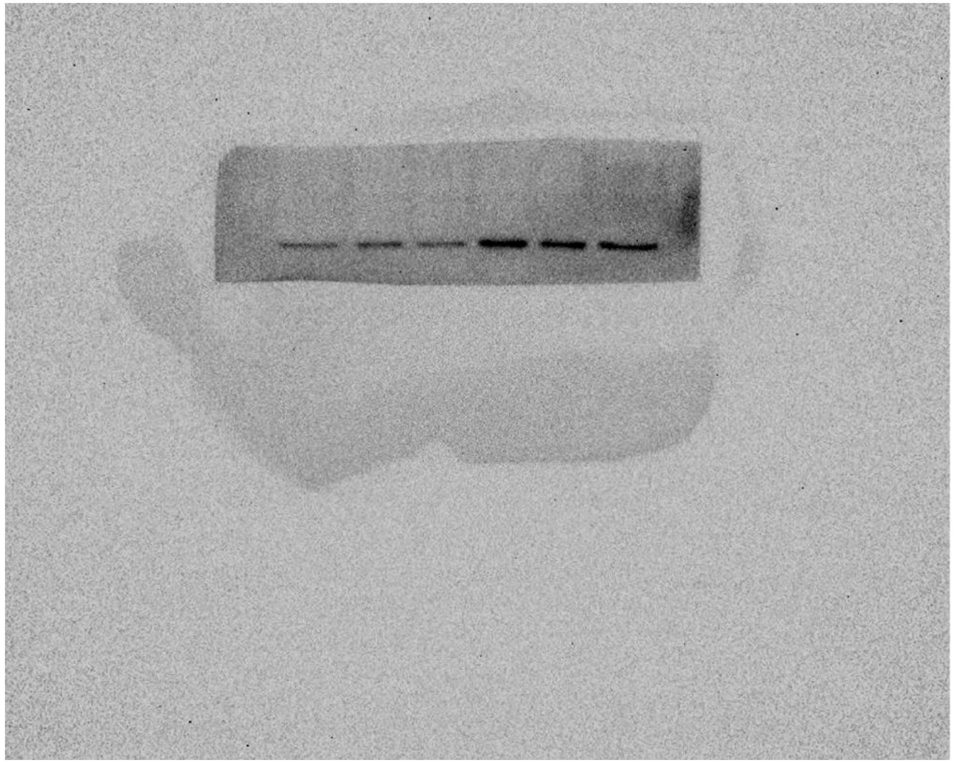

p38 MAPK

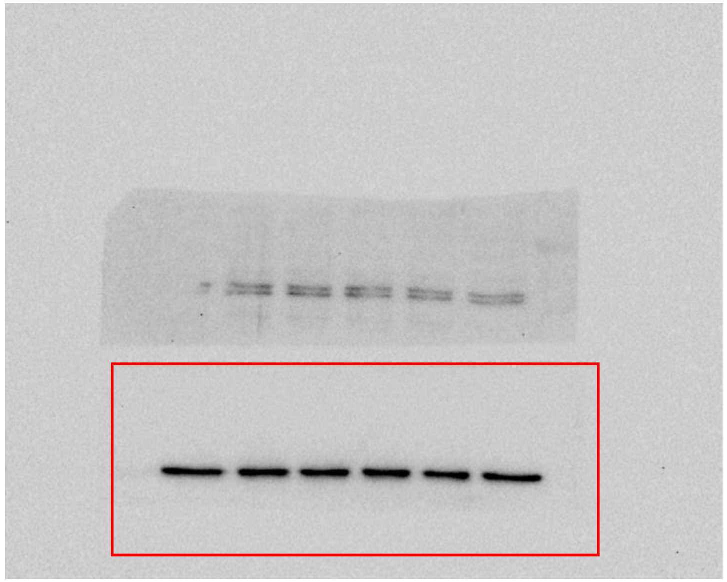

Fig.2J

β1AR

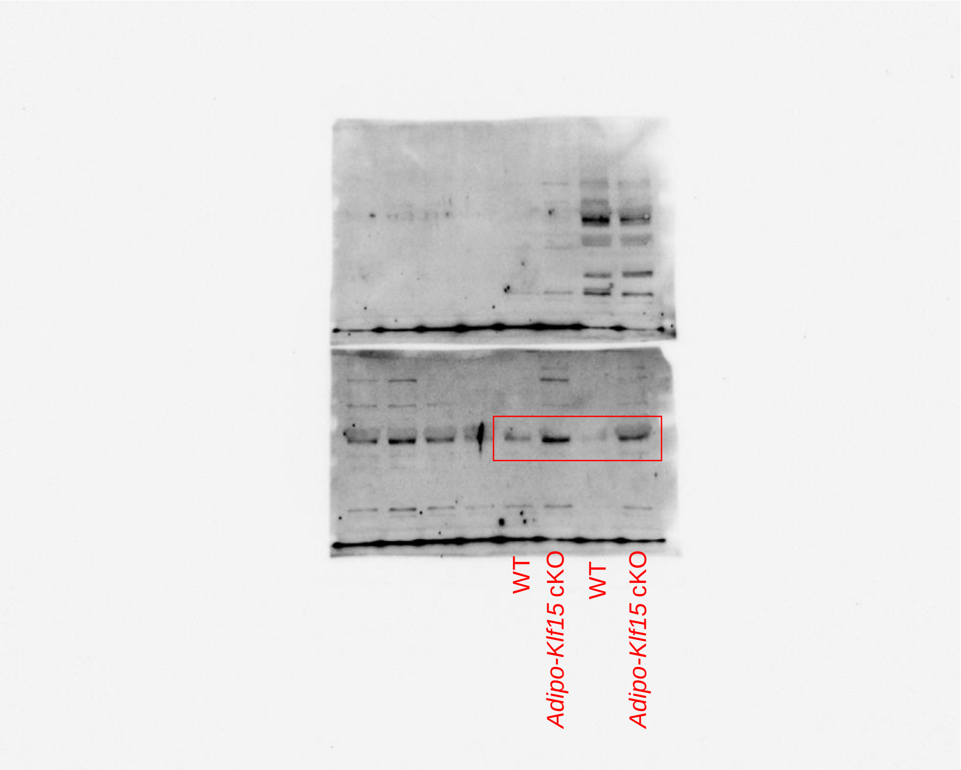

βactin

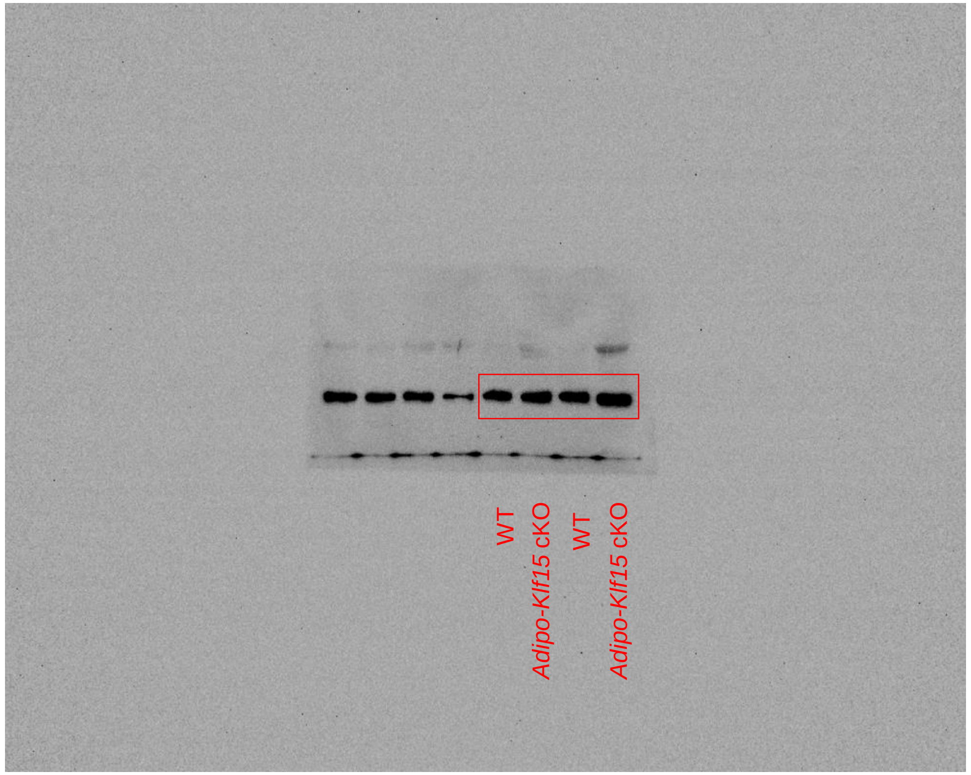

Fig.3K

UCP1

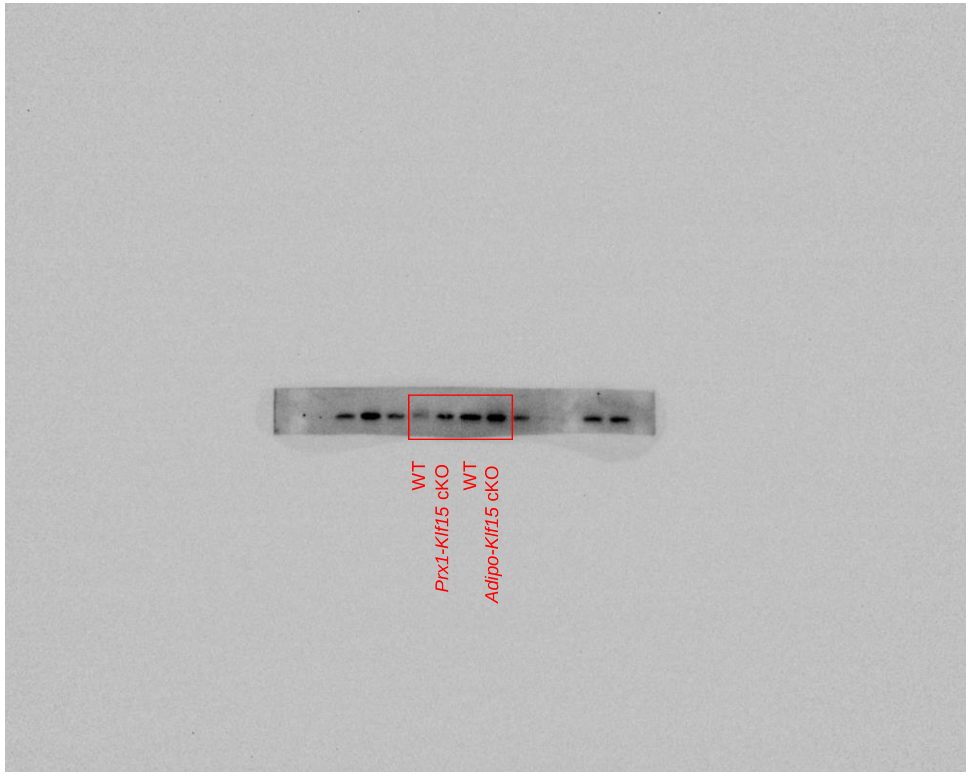

GAPDH

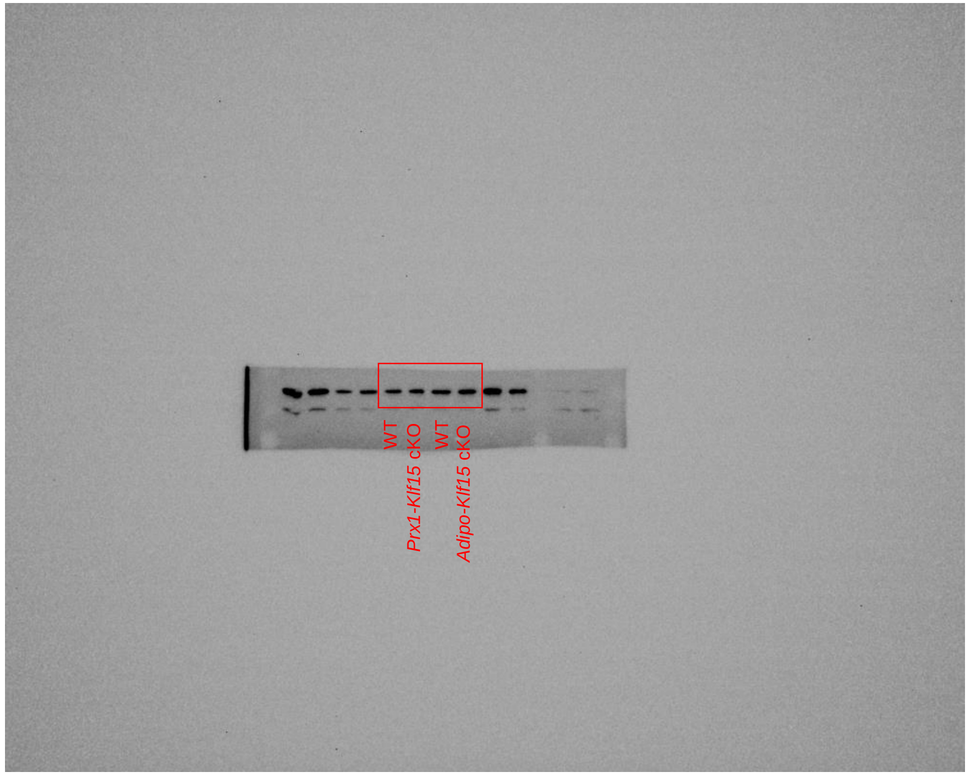

Fig.4H

ChIP-PCR gel

Primer 2 for target region

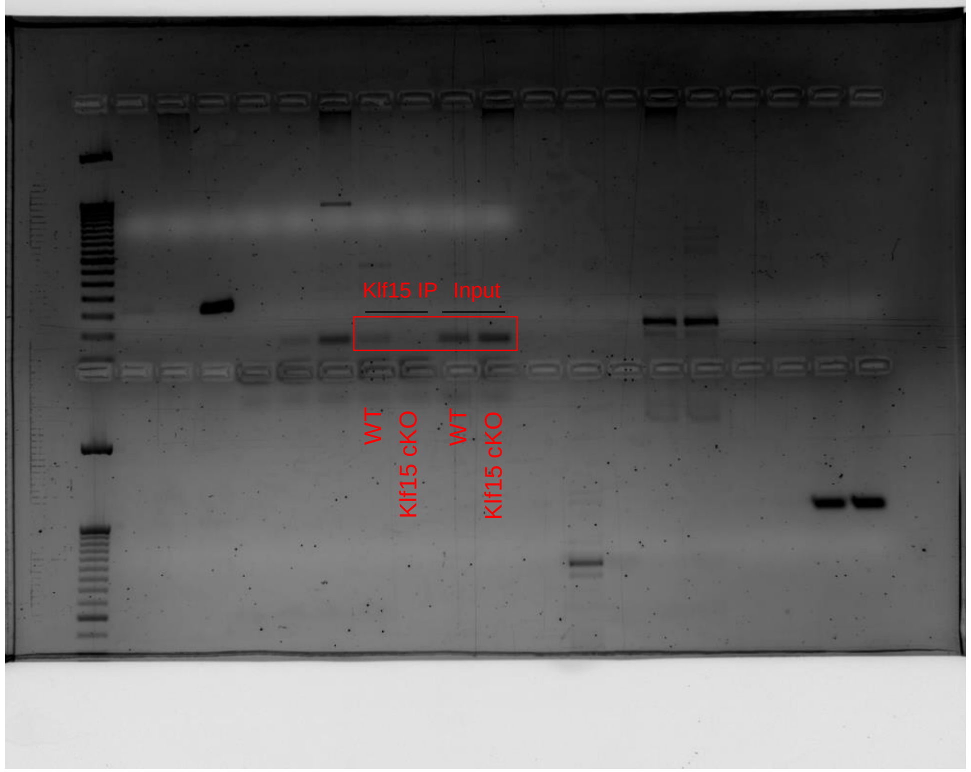

Negative primer for control region

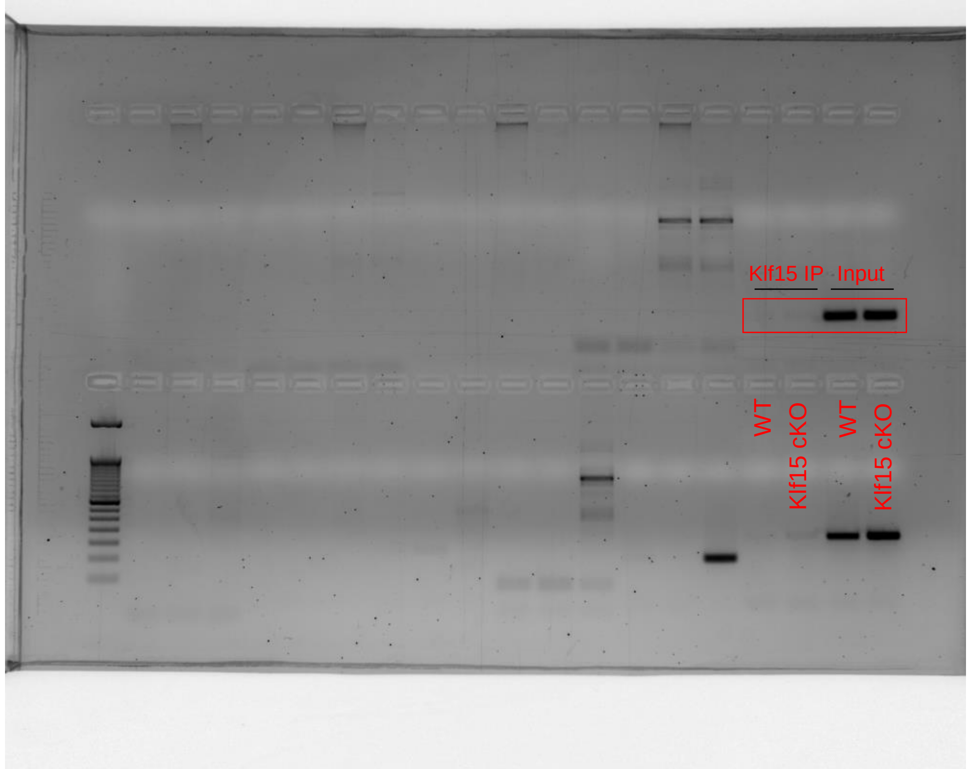

Supplemental Fig.1B

β1AR

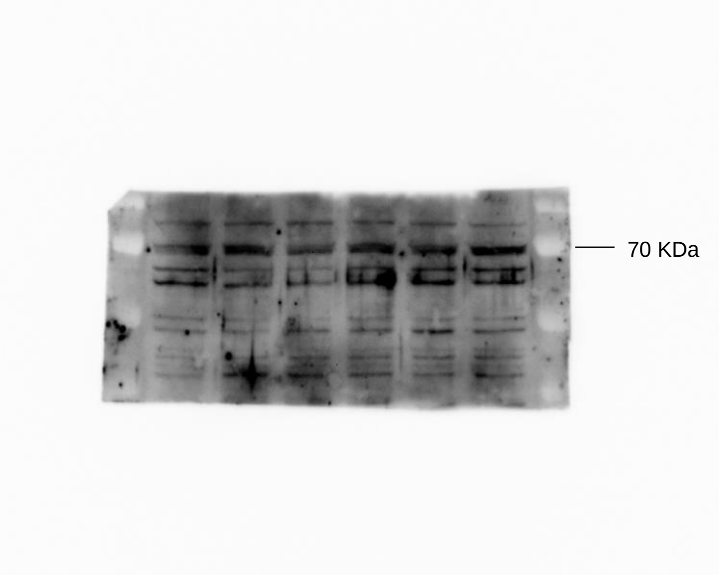

70 KDa

β-Tubulin

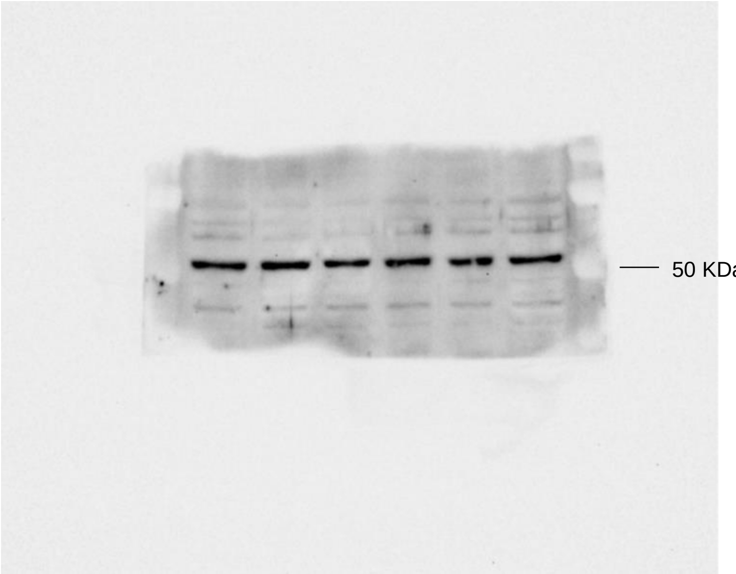

50 KDa

Supplemental Fig.2F

β1AR

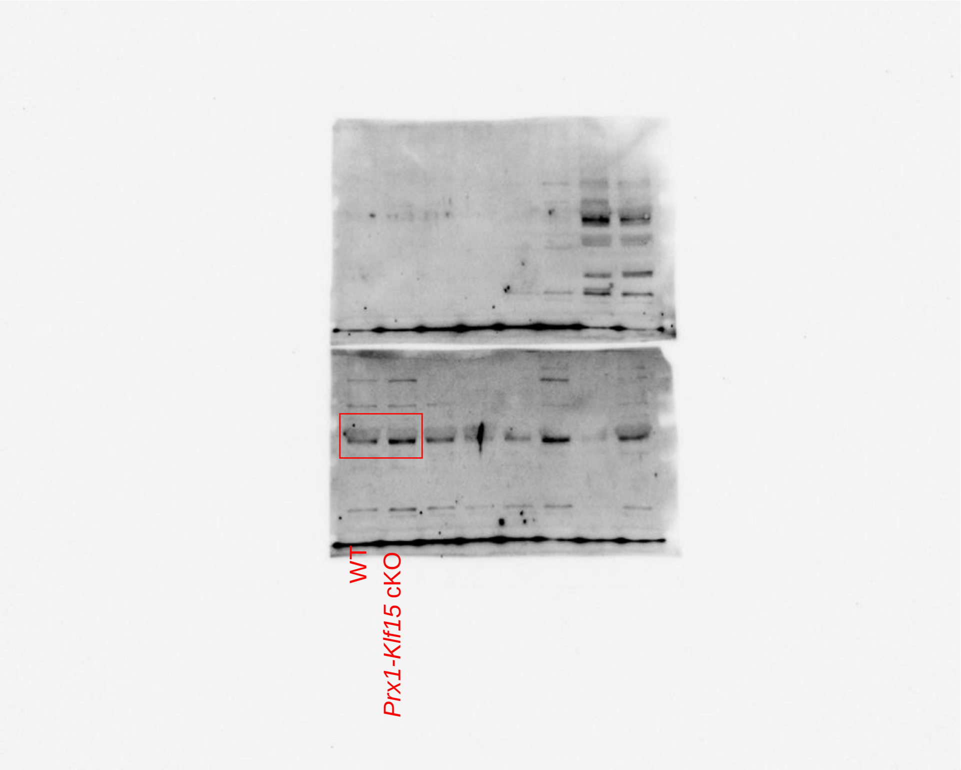

βactin

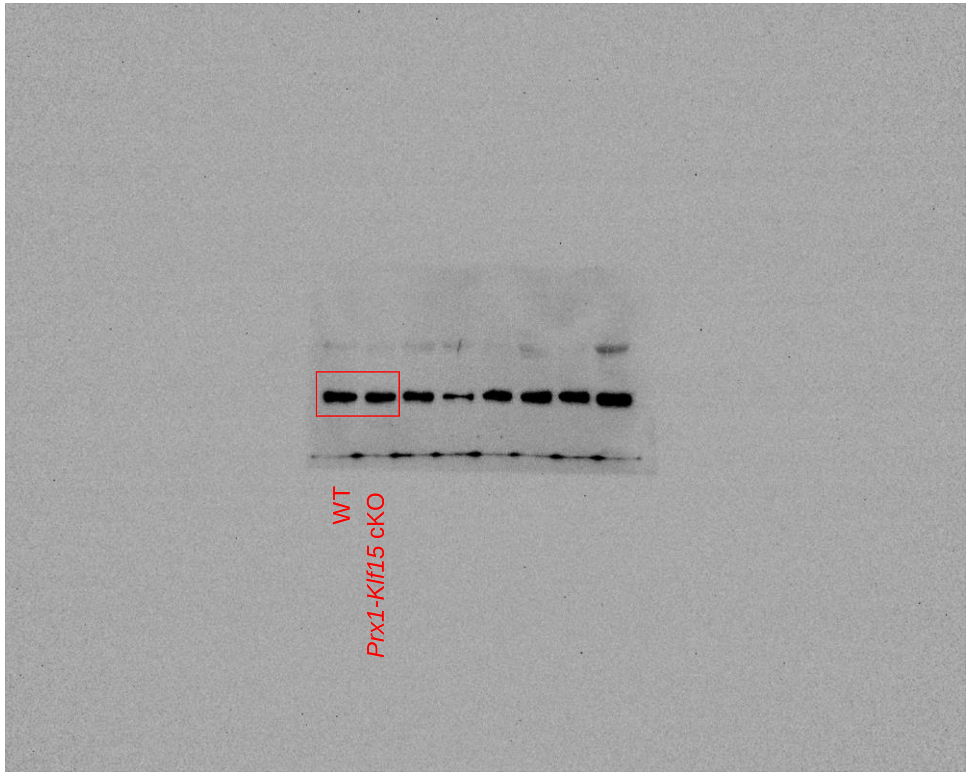

Supplemental Fig.4F

β1AR

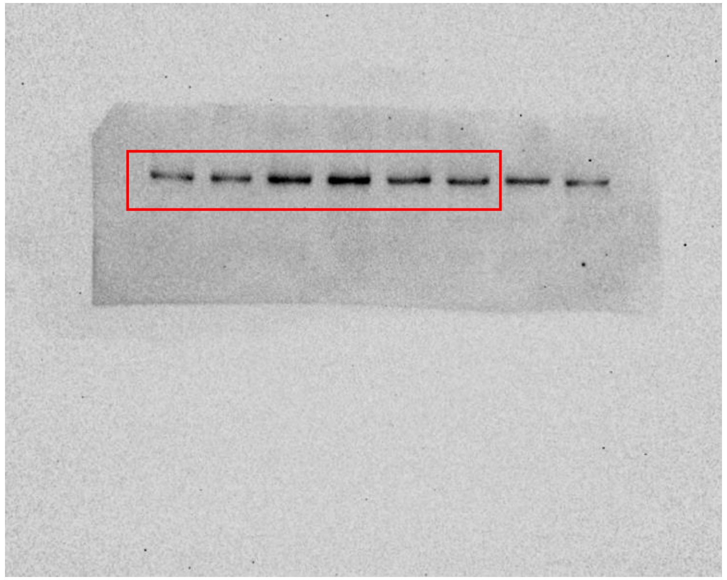

GAPDH

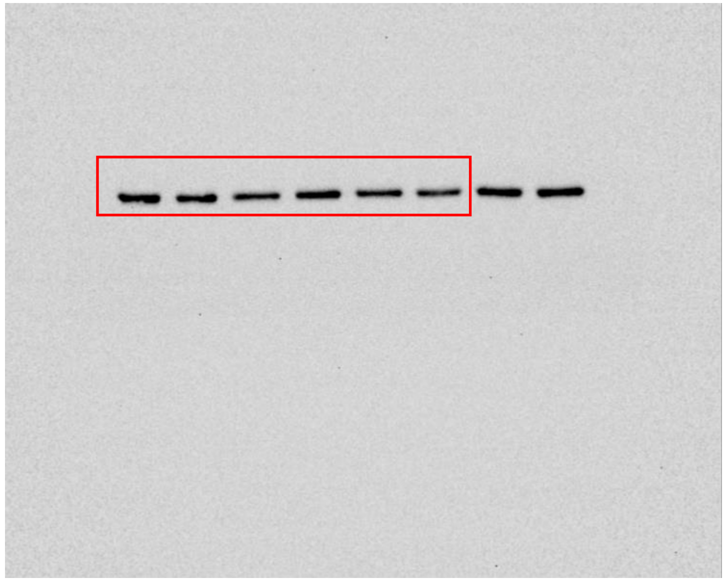

Supplement: Unedited blot and gel images [file jci-134-172360-s190.pdf]
